# Sorting Phenomena and Chirality Transfer in Fluoride-Bridged Macrocyclic Rare Earth Complexes

**DOI:** 10.1021/acs.inorgchem.1c03034

**Published:** 2021-11-16

**Authors:** Katarzyna Ślepokura, Trevor A. Cabreros, Gilles Muller, Jerzy Lisowski

**Affiliations:** † Department of Chemistry, University of Wrocław, 14 F. Joliot-Curie, 50-383 Wrocław, Poland; ‡ Department of Chemistry, San José State University, One Washington Square, San José, California 95192-0101, United States

## Abstract

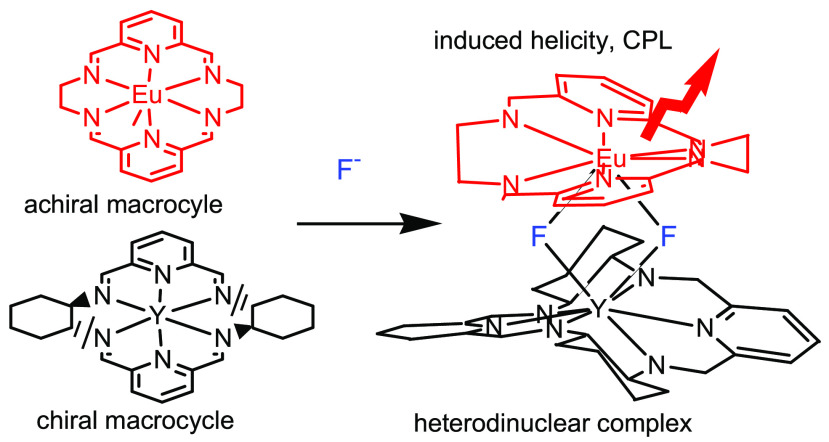

The reaction of fluoride anions with mononuclear lanthanide(III) and yttrium(III) hexaaza-macrocyclic complexes results in the formation of dinuclear fluoride-bridged complexes. As indicated by X-ray crystal structures, in these complexes two metal ions bound by the macrocycles are linked by two or three bridging fluoride anions, depending on the type of the macrocycle. In the case of the chiral hexaaza-macrocycle L1 derived from *trans*-1,2-diaminocyclohexane, the formation of these μ_2_-fluorido dinuclear complexes is accompanied by enantiomeric self-recognition of macrocyclic units. In contrast, this kind of recognition is not observed in the case of complexes of the chiral macrocycle L2 derived from 1,2-diphenylethylenediamine. The reaction of fluoride with a mixture of mononuclear complexes of L1 and L2, containing two different Ln(III) ions, results in narcissistic sorting of macrocyclic units. Conversely, a similar reaction involving mononuclear complexes of L1 and complexes of achiral macrocycle L3 based on ethylenediamine results in sociable sorting of macrocyclic units and preferable formation of heterodinuclear complexes. In addition, formation of these heterodinuclear complexes is accompanied by chirality transfer from the chiral macrocycle L1 to the achiral macrocycle L3 as indicated by CPL and CD spectra.

## Abstract 2

Dinuclear lanthanide(III) and yttrium(III) complexes were obtained by linking hexaza-macrocyclic units with two or three bridging fluorides. When two different macrocyclic complexes are used as substrates, the formation of dinuclear species is accompanied by social or narcissistic sorting of macrocyclic units depending on the kind of the macrocycle. An achiral macrocycle within such a mixed dimer assumes a preferred direction of helical twist caused by the steric interaction with a chiral macrocyclic partner.

## Introduction

Chiral structures and chiral recognition phenomena are fundamental features of molecular biological systems, and chirality is a central issue in various areas of organic and inorganic chemistry. For instance, chiral metal complexes and chiral supramolecular assemblies are studied as enantioselective catalysts, chiroptical probes, and nonlinear optical materials. Similarly, the recognition and self-organization phenomena characteristic for complex biological systems have triggered research in many areas of chemistry. Both social self-sorting (self-discrimination) and narcissistic self-sorting (self-recognition) are examples of such phenomena that attract increasing attention.^1−33^

Chiral sorting corresponds to enantiomeric self-recognition or enantiomeric self-discrimination, and these processes have been documented for supramolecular systems,^1−6^ metal complexes,^7−21^ and organic systems, including macrocyclic compounds.^22−31^ Chiral sorting phenomena are most often demonstrated for solid state, while examples of enantiomeric self-recognition well documented for solutions of metal complexes are less common. Another important issue in the synthesis of elaborate enantiopure metal complexes or supramolecular assemblies is chirality transfer,^32−44^ e.g., the transmission of chiral information from enantiopure ligands to metal centers. While there are many chiral transition metal complexes with well-defined stable configurations, the control over chirality of lanthanide complexes, in particular polynuclear complexes,^35−37,45−59^ is more difficult due to the lack of spatial preferences, lability, and high-coordination numbers of these ions. For similar reasons recognition and self-sorting phenomena^4,6−12,20,21^ in lanthanide systems are not so well explored in comparison with the systems based on stable organic compounds or more rigid and inert transition metal complexes.

**Figure 1:**
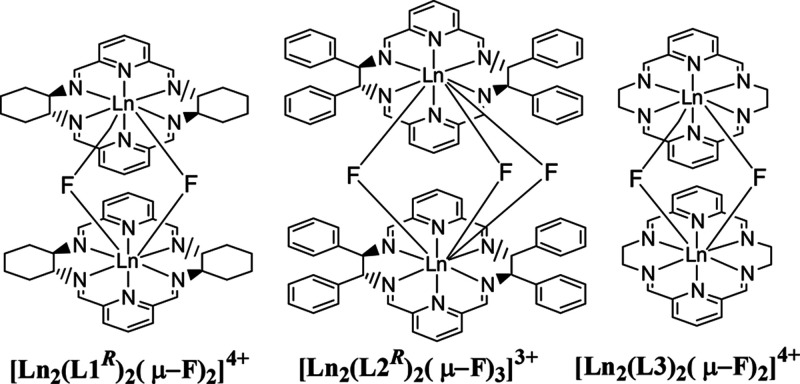
Macrocycles L1–L3 in their dinuclear lanthanide(III) complexes (axial ligands omitted for simplicity).

Here we describe fluoride derivatives of lanthanide(III) and Y(III) (denoted as Ln(III)) complexes of hexaaza-macrocycles L1–L3 derived from 1,2-diformylpyridine and various diamines (Figure 1). We show that dinuclear complexes of this type may contain two different macrocyclic units and that their formation is governed by self-sorting phenomena. By using circularly polarized luminescence (CPL) and circular dichroism (CD) spectroscopy, we also demonstrate chirality transfer from chiral to achiral macrocycle in these mixed dinuclear complexes.

The number of well-defined molecular lanthanide(III) complexes containing fluoride ligands is limited due to the tendency to precipitate insoluble Ln(III) fluoride salts as well as due to the difficulty in controlling the coordination sphere of these labile ions.^60−73^ In particular, macrocyclic ligands based on tetraaza-cyclen framework strongly bind lanthanide(III) ions and form stable fluoride derivatives. Some of these cyclen-based complexes are mononuclear and contain terminal fluoride anions,^67−69^ while other are dinuclear where two macrocyclic units are linked by a single linear μ_2_-fluorido bridge.^70−73^ The binding of fluoride by these cyclen-based Eu(III) and Tb(III) complexes has been studied in the context of sensing of fluoride anions by using luminescence spectroscopy. In addition, a terminal fluoride anion bound in the axial position in cyclen-based Dy(III) complexes and in polychelate Dy(III) complexes generates high magnetic anisotropy of the Dy(III) ions and enhances single-ion magnet (SIM) properties. It has been also suggested that the Dy(III) complex with a hexaaza-macrocycle derived from 1,2-diacetylpyridine and ethylenediamine should exhibit exceptional magnetic anisotropy and SIM behavior.^74^

CPL, the emission analogue to CD, involves the emission of circularly polarized luminescence from a chiral compound.^75−84^ Unlike CD spectroscopy, CPL is only dependent on the active CPL species and free of potentially interfering background signals. It must be noted that a combination of positive and negative CPL signs ensures the splitting of narrow emission lines of the Ln^3+^ ions, which provide unique chiroptical properties that can be used to probe for chiral phenomena. Thus, the CPL activity typically acts as a “fingerprint” to indicate any structural changes within the Ln(III)-containing system and/or around the local environment of the Ln(III) metal.

## Results and Discussion

### Mononuclear Lanthanide(III) Complexes of Macrocycle L2

**Figure 2:**
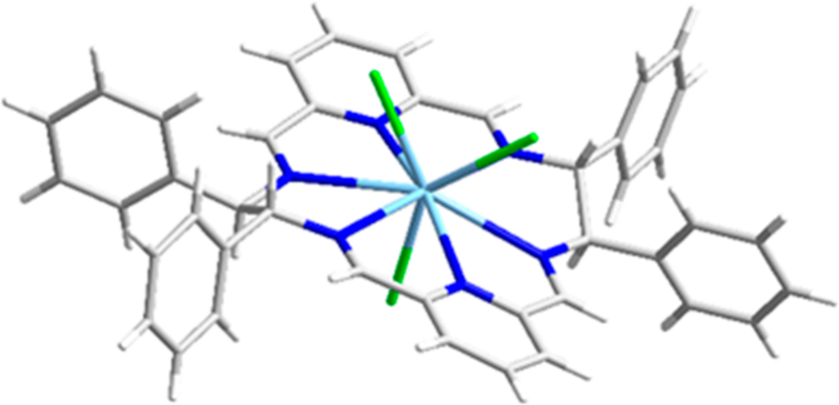
Crystal structure of the [La(L2^*R*^)Cl_3_] complex in the [La(L2^*R*^)Cl_3_]·2.5MeOH·0.5H_2_O crystal. Gray: C atoms; dark blue: N; green: Cl; light blue: La.

**Figure 3:**
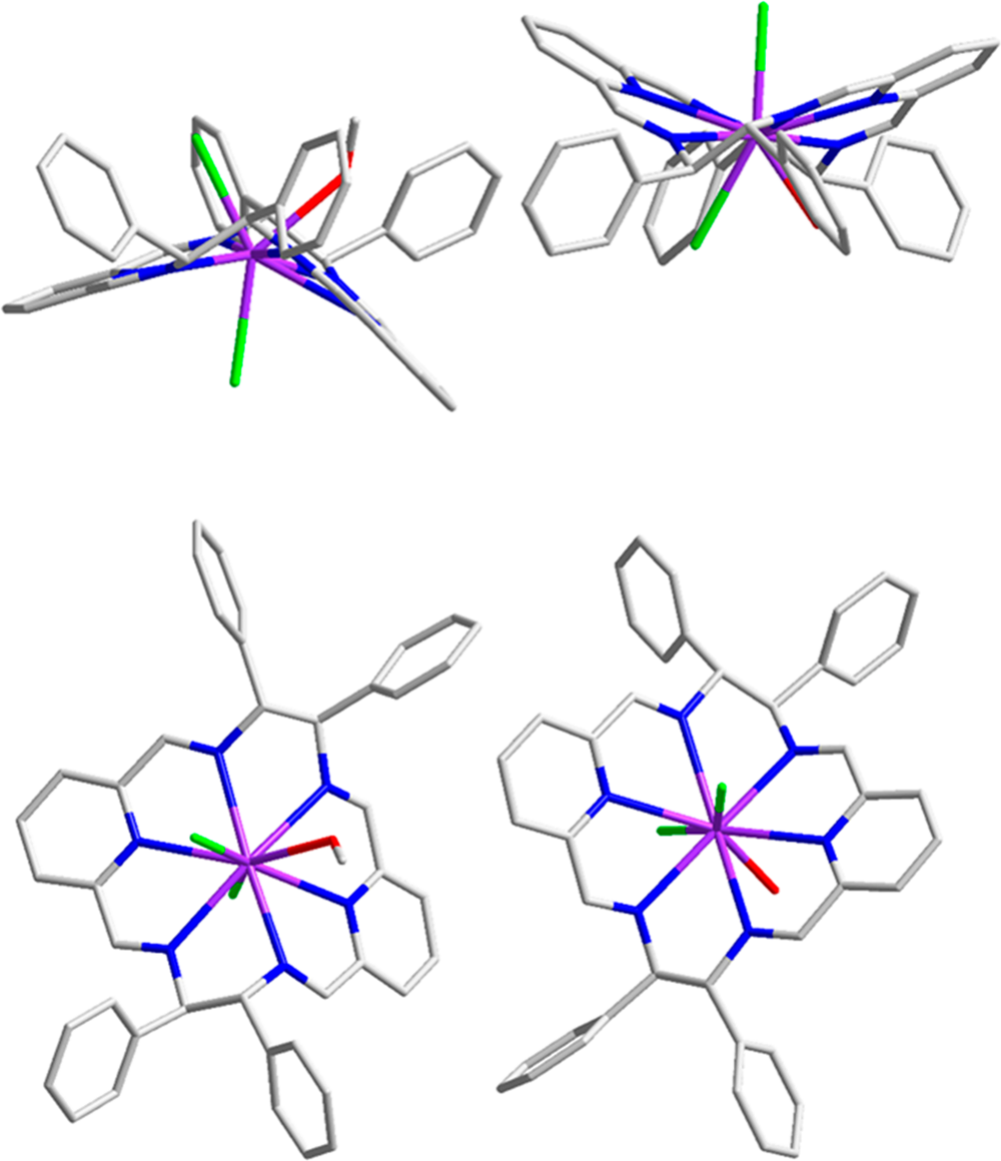
Side and top views of the complex cations [Tb(L2^*R*^)Cl_2_(MeOH)]^+^ and [Tb(L2^*R*^)Cl_2_(H_2_O)]^+^ of the {[Tb(L2^*R*^)Cl_2_(MeOH)][Tb(L2^*R*^)Cl_2_(H_2_O)]}Cl_2_·9MeOH·H_2_O crystal (hydrogen atoms omitted for clarity). Gray: C atoms; blue: N; green: Cl; red: O; violet: Tb.

The new enantiopure rare earth(III) complexes of macrocycle L2 have been obtained in a template synthesis from the lanthanide(III) (Ln = Pr, Nd, Tb) or yttrium(III) chlorides, 2,6-diformylpyridine, and (1*R*,2*R*)-1,2-diphenylethylenediamine or (1*S*,2*S*)-1,2-diphenylethylenediamine in the same manner as it was reported for La, Eu, and Dy complexes.^85,86^ The crystal structure of the [La(L2^*R*^)Cl_3_]·2.5MeOH·0.5H_2_O complex, isomorphic to the previously reported Ce(III) derivative,^85^ shows 9-coordinate La(III) ion bound by the six nitrogen atoms of the macrocycle and three axial chloride anions (Figure 2). The macrocycle L2 is relatively flat in this complex with moderate helical twist of the pyridine fragments and very small folding of the macrocycle reflected by almost linear arrangement of the two pyridine nitrogen atoms and the central metal ion. In contrast, in the related Tb(III) complex the macrocycle is not only helically twisted but also sizably folded (Figure 3). The asymmetric unit of the {[Tb(L2^*R*^)Cl_2_(MeOH)][Tb(L2^*R*^)Cl_2_(H_2_O)]}Cl_2_·9MeOH·H_2_O crystal contains two different cationic complexes [Tb(L2^*R*^)Cl_2_(MeOH)]^+^ and [Tb(L2^*R*^)Cl_2_(H_2_O)]^+^. Both cations contain nine-coordinate Tb(III) ions. The overall structures of these two cations are similar, but they differ in the set of axial ligands—one of them contains two axial chloride anions and coordinated water molecule, while the other contains two axial chloride anions and coordinated methanol molecule. Unlike the La(III) case, the macrocycle L2 is considerably folded in its Tb(III) complex similarly as it was observed for the Eu(III) complex^85^ and the recently reported Dy(III) complex.^86^

The NMR spectra of the Pr(III), Nd(III), Tb(III), and Dy(III) complexes of L2 cover a wide range of chemical shifts and show very broad lines (in particular in the case of Tb(III) and Dy(III) derivatives) in accord with the binding of the paramagnetic metal ion in the center of the macrocycle. The ^1^H NMR spectra of the [Ln(L2)Cl_3_] complexes consist of seven signals of the ligand L2. This number of lines indicates an effective *D*_2_ symmetry of the complexes reflecting dynamic averaging of the structures observed in the crystalline state. This process most likely results from fast axial ligand exchange on the NMR time scale.

**Figure 4:**
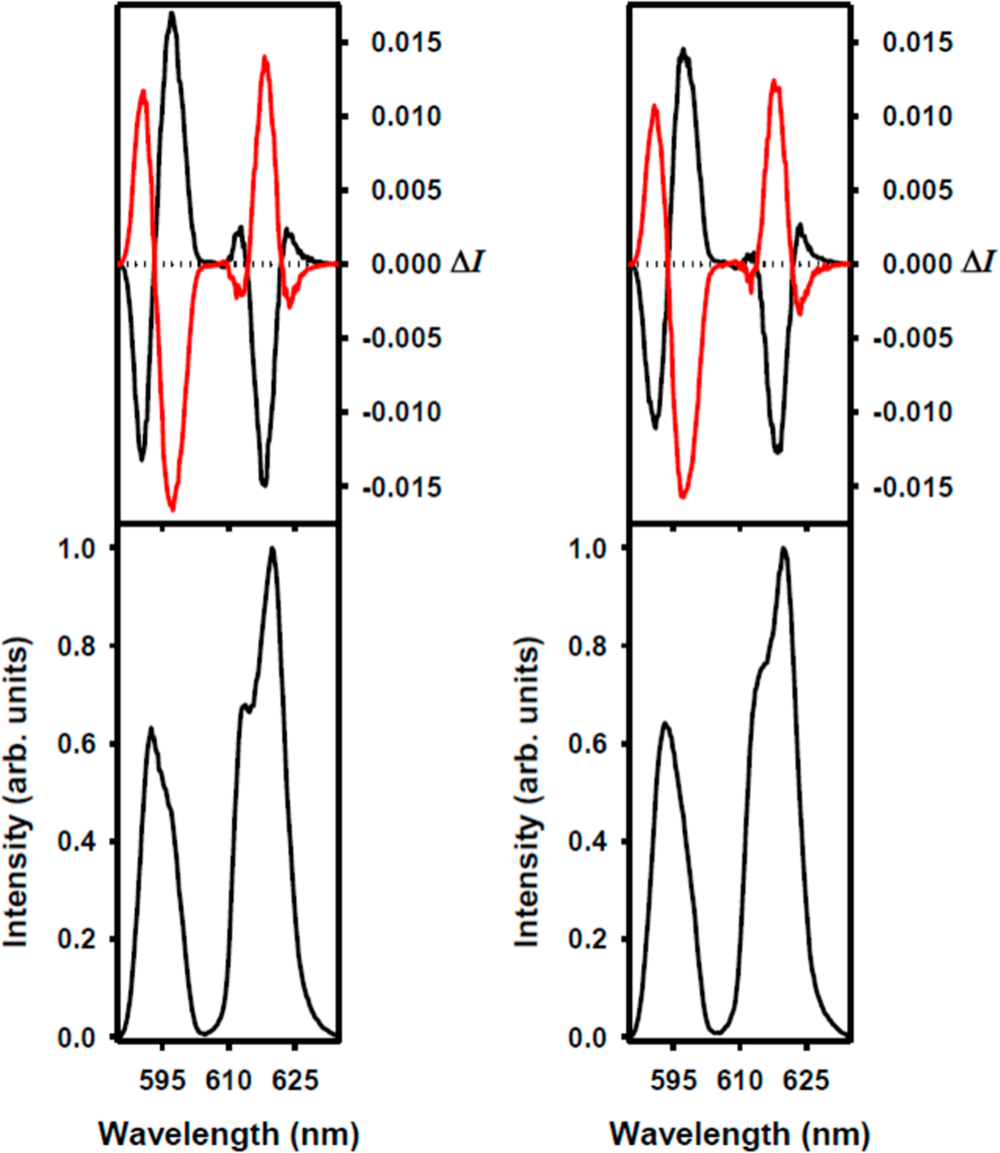
CPL (upper curves) and total luminescence (lower curves) spectra for the ^5^D_0_ → ^7^F_1_ and ^5^D_0_ → ^7^F_2_ transitions of [Eu(L2^*S*^)Cl_3_] (black) and [Eu(L2^*R*^)Cl_3_] (red) in 1 mM nondeuterated (left) and deuterated (right) 2:1 chloroform:methanol at 295 K, upon excitation at 333/329 and 329/330 nm, respectively.

Because the [Ln(L2)Cl_3_] complexes can be obtained in enantiopure form and Eu(III) and Tb(III) complexes may be luminescent, we were interested in CPL activity of the complexes of the L2^*R*^ and L2^*S*^ optical isomers of the macrocycle. The CPL measurements were performed in nondeuterated and deuterated 2:1 chloroform/methanol solutions at concentrations of 1 mM. The transitions that we studied are the magnetic dipole allowed transitions, ^5^D_0_ → ^7^F_1_ for Eu(III) and ^5^D_4_ → ^7^F_5_ for Tb(III), where one predicts the CPL would be large. We were able to measure a CPL signal for the set of enantiomeric pairs of the chiral Eu(III)-containing compounds upon UV excitation (Figure 4). The luminescence dissymmetry ratio, *g*_lum_, is defined as follows:
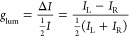
where *I*_L_ and *I*_R_ refer respectively to the intensity of left and right circularly polarized light.

For the pair of the Eu(III) complexes [Eu(L2^*R*^)Cl_3_] and [Eu(L2^*S*^)Cl_3_], we were able to record opposite CPL spectra, which show the Eu(III)-centered polarized emission following excitation at about 333–329 nm (nondeuterated solutions) and 329–330 nm (deuterated solutions), respectively. The transition that we studied is the magnetic dipole allowed transitions, ^5^D_0_ → ^7^F_1_ for Eu(III). In addition, we also recorded the CPL activity for the (Eu) ^5^D_0_ → ^7^F_2_. The CPL activity observed from the two enantiomeric forms of the Eu(III)-containing complexes is roughly similar, with a magnitude of the *g*_lum_ values a little smaller for the samples measured in deuterated versus nondeuterated 2:1 chloroform/methanol solutions. The *g*_lum_ values are −0.05/+0.05, +0.05/–0.05, −0.03/+0.03 versus −0.05/+0.05, +0.07/–0.06, −0.03/+0.04 for the three components (∼591, 596, and 618 nm) of the CPL spectra for the Eu(III) complexes of the L2^*S*^/L2^*R*^ enantiomers of the macrocycle, respectively (Figure 4).

It was not possible to record the CPL activity of the two enantiomeric forms of the Tb(III)-containing complex L2 in nondeuterated and deuterated 2:1 chloroform/methanol solutions at concentrations of 1 and 10 mM. The intensity was too weak, suggesting that there is most likely an effective back-transfer from the Tb(III) to the ligand and/or an inefficient intersystem crossing between the singlet and triplet states of the ligand taking place for the Tb(III)-containing complexes. However, the observation of the CPL activity for the Eu(III)-containing compounds tends to favor an efficient Tb(III) back-transfer phenomenon.^87^

### Crystal Structures of Fluoride-Bridged Homodinuclear Ln(III) Complexes of Macrocycles L1–L3

**Figure 5:**
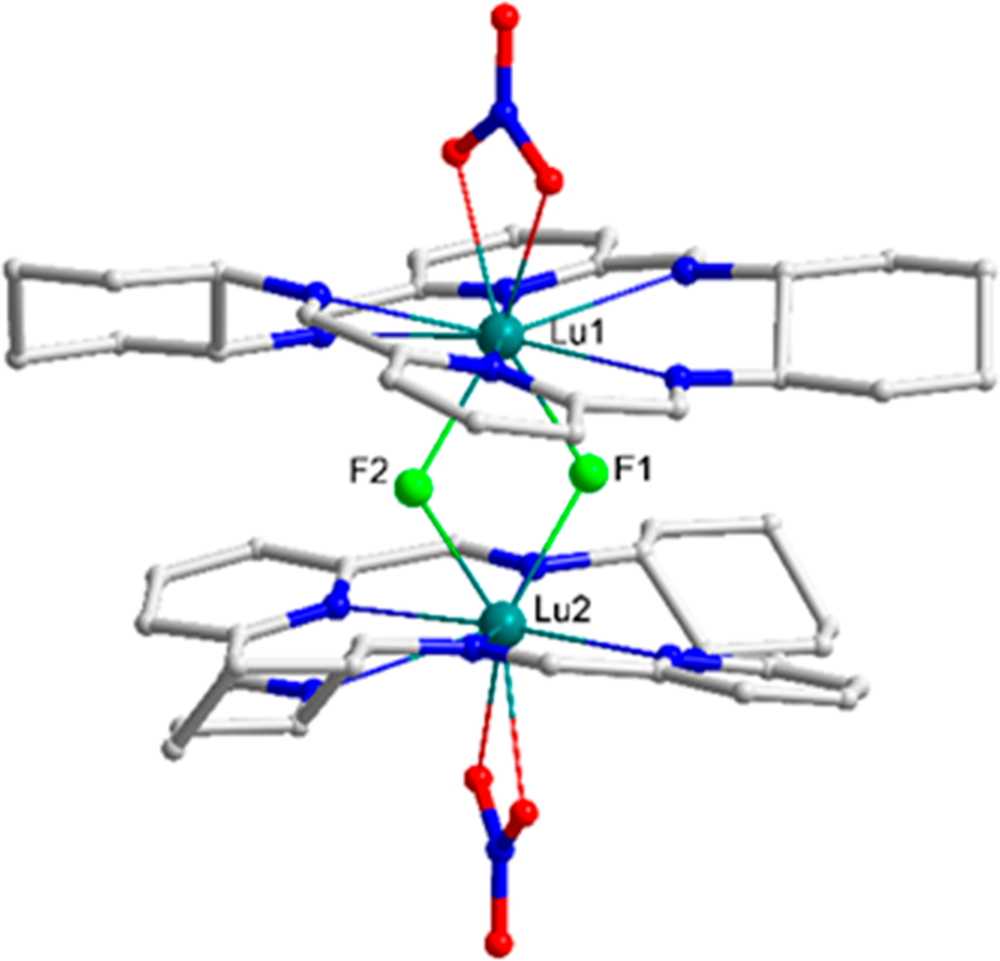
Side view of the dimeric cationic complex [Lu_2_(L1^*R*^)_2_(μ_2_-F)_2_(NO_3_)_2_]^2+^ present in the crystal of [Lu_2_(L1^*R*^)_2_(μ_2_-F)_2_(NO_3_)_2_](NO_3_)_2_·CHCl_3_·3H_2_O (hydrogen atoms omitted for clarity). Gray: C atoms; blue: N; green: F; red: O; teal: Lu.

The dimeric [Ln_2_(L1)_2_(μ_2_-F)_2_(NO_3_)_2_](NO_3_)_2_ complexes have been obtained from the monomeric nitrate derivatives [Ln(L1)(NO_3_)_2_](NO_3_) by addition of a stoichiometric amount of potassium fluoride or tetraethylamonium fluoride, NEt_4_F. The crystal structure of the Lu(III) complex [Lu_2_(L1^*R*^)_2_(μ_2_-F)_2_(NO_3_)_2_](NO_3_)_2_·CHCl_3_·3H_2_O (Figure 5, Figures S1 and S2) indicates two parallel macrocyclic units linked by two bridging fluoride anions. The crystal of this compound contains the cationic complex [Lu_2_(L1^*R*^)_2_(μ_2_-F)_2_(NO_3_)_2_]^2+^, free nitrate counterions, and solvent molecules. In the [Lu_2_(L1^*R*^)_2_(μ_2_-F)_2_(NO_3_)_2_]^2+^ dimers the ten-coordinate Lu(III) cation is bound in equatorial positions by six nitrogen atoms of the chiral macrocycle L1, while the axial positions are occupied by two bridging fluoride anions and a bidentate nitrate anion. The overall structure of this cationic complex is similar to the structure of the analogous hydroxo-bridged Nd(III) cationic complex [Nd_2_(L1^*S*^)_2_(μ_2_-OH)_2_(NO_3_)_2_]^2+^.^21^ The macrocyclic ligand L1 in these complexes is helically twisted, and the direction of the helical twist is determined by the configuration at the asymmetric carbon atoms of the diaminocyclohexane units. Thus, the twist of the two pyridine rings of L1 corresponding to the mutual Δ orientation is associated with the L1^*S*^ enantiomer of the ligand, while the opposite Λ twist of these units is associated with the L1^*R*^ enantiomer.

The preliminary structural data for the [Dy_2_(L1)_2_(μ_2_-F)_2_(NO_3_)_2_](NO_3_)_2_·CHCl_3_·*n*H_2_O crystal show that its structure is very similar (isostructural) to the above Lu(III) complex (Figure S3). The structure of Eu(III) complex of [Ln_2_(L1)_2_(μ_2_-F)_2_(NO_3_)_2_](NO_3_)_2_ type could not be satisfactorily solved due to crystal quality; however, the crude model shows its isostructurality to the Lu(III) crystal.

**Figure 6:**
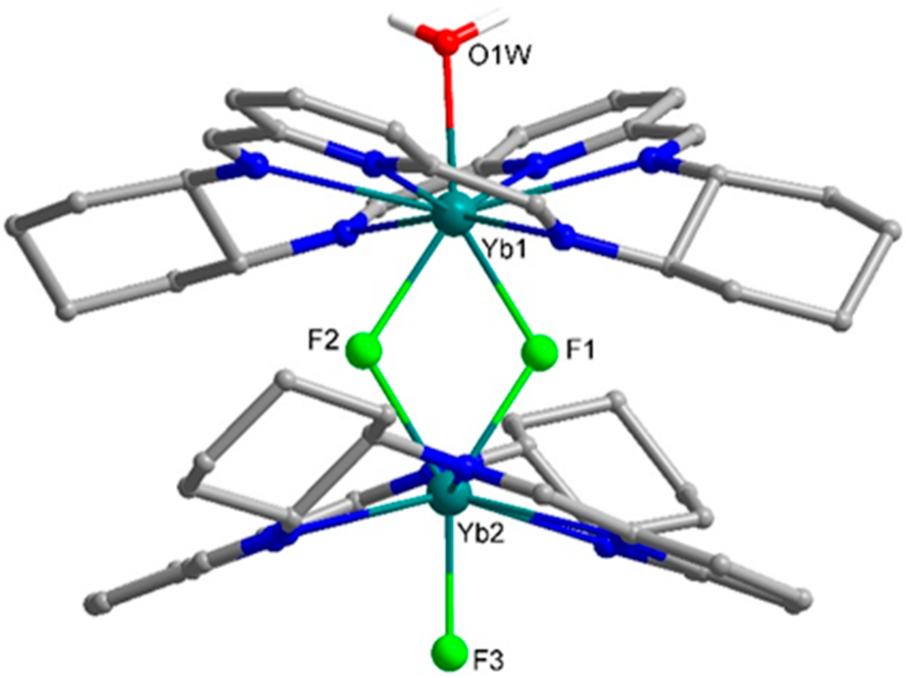
Dinuclear cationic complex [Yb_2_(L1^*R*^)_2_(μ_2_-F)_2_F(H_2_O)]^3+^ present in the crystals of [Yb_2_(L1^*R*^)_2_(μ_2_-F)_2_F(H_2_O)]Cl_3_·3.5MeOH·4.5H_2_O (hydrogen atoms, except water, omitted for clarity). Gray: C atoms; blue: N; green: F; red: O; teal: Yb.

Similar fluoride-bridged dinuclear complexes can be obtained from the monomeric chloride derivatives [Ln(L1)Cl_3_]. For instance, the reaction of Yb(III) complex of this type with 1.5 equiv of NEt_4_F results in isolation of crystals of the [Yb_2_(L1^*R*^)_2_(μ_2_-F)_2_F(H_2_O)]Cl_3_·3.5MeOH·4.5H_2_O complex where one of the Yb(III) ions contains additional terminal fluoride anion in the axial position, while the other Yb(III) contains an axial water molecule (Figure 6 and Figure S4). In the crystal lattice of this complex the complex cation [Yb_2_(L1^*R*^)_2_(μ_2_-F)_2_F(H_2_O)]^3+^ is linked by hydrogen bonds linking the fluoride and axial water molecules belonging to different complex cations, which leads to formation of supramolecular helical chains (Figure S5).

**Figure 7:**
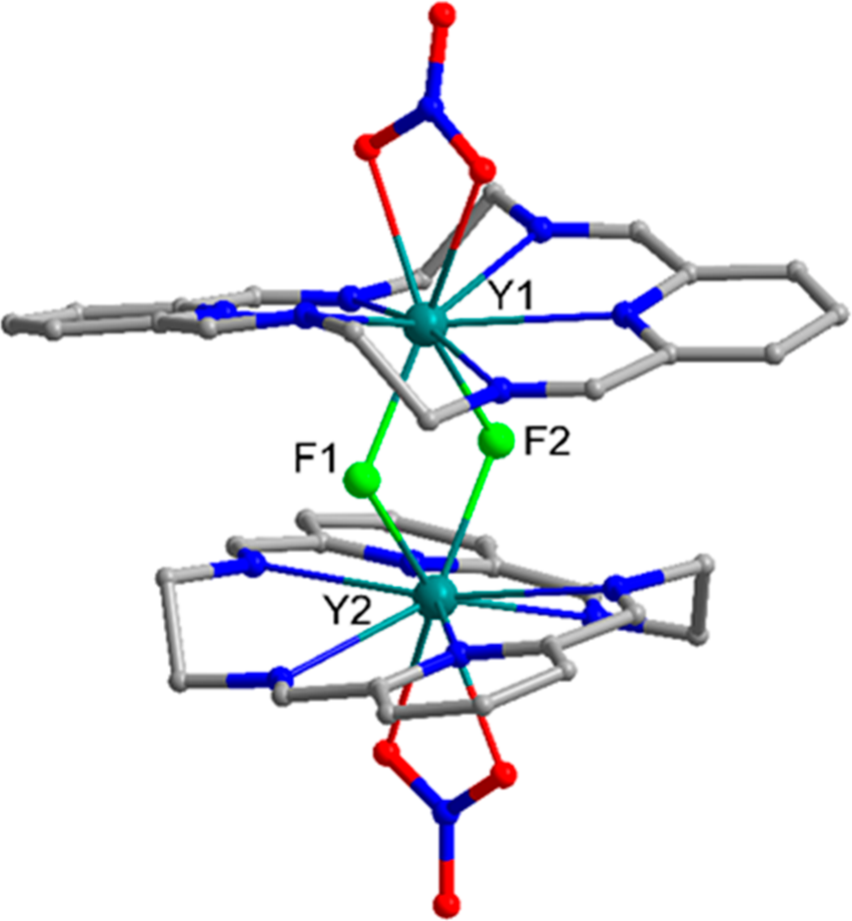
Side view of the dimeric cationic complex [Y_2_(L3)_2_(μ_2_-F)_2_(NO_3_)_2_]^2+^, present in the crystal of [Y_2_(L3)_2_(μ_2_*-*F)_2_(NO_3_)_2_](NO_3_)_2_·CHCl_3_·MeOH·H_2_O (hydrogen atoms omitted for clarity). Gray: C atoms; blue: N; green: F; red: O; teal: Y.

Dimeric fluorido-bridged complexes are also formed in the reactions of mononuclear nitrate-type complexes [Ln(L3)(NO_3_)_2_](NO_3_) of the macrocycle L3 derived from ethylenediamine. The molecular structure of the cationic complex [Y_2_(L3)_2_(μ_2_-F)_2_(NO_3_)_2_]^2+^, present in the crystal structure of the dimeric Y(III) complex of L3, is analogous to the above-discussed complexes of L1 (Figure 7 and Figure S6). The conformations of the chiral macrocycle L1 and the achiral macrocycle L3 in these fluoride-bridged complexes are similar. Importantly, the achiral ligand L3 is helically twisted in the complexed form, and thus, it assumes a chiral conformation. Within the fluorido-bridged complex [Y_2_(L3)_2_(μ_2_-F)_2_(NO_3_)_2_](NO_3_)_2_·CHCl_3_·MeOH·H_2_O both macrocyclic units adopt the same direction of helical twist, either ΔΔ or ΛΛ, and the centrosymmetric crystals of this complex contain both forms as a racemic mixture.

**Figure 8:**
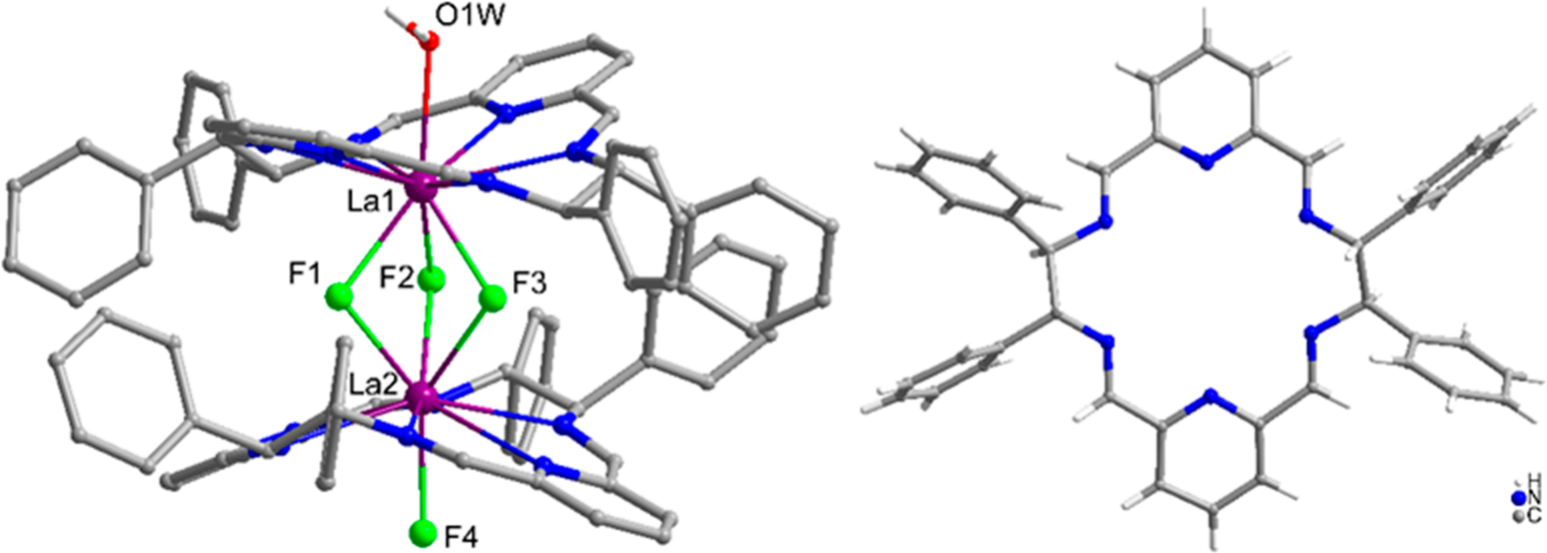
Left: side view of the [La_2_(L2^*R*^)_2_(μ_2_-F)_3_F(H_2_O)]^2+^ cation present in the [La_2_(L2^*R*^)_2_(μ_2_-F)_3_F(H_2_O)]Cl_2_·5MeOH·H_2_O crystal (hydrogen atoms, except water, and the disorder of bridging fluoride anions and two phenyl rings omitted for clarity). Right: top view of the macrocycle L2 in this complex. Gray: C atoms; blue: N; green: F; red: O; plum: La.

The reaction of the complex of the macrocycle L2, [La(L2^*R*^)Cl_3_], with NEt_4_F results in a different kind of dimer in comparison with the dimeric complexes of macrocycles L1 and L3 discussed above. Thus, the X-ray crystal structure of the obtained product [La_2_(L2^*R*^)_2_(μ_2_-F)_3_F(H_2_O)]Cl_2_·5MeOH·H_2_O shows a dimeric structure where three fluoride anions bridge the La(III) ions (Figure 8 and Figure S7). In contrast, in the dimeric Ln(III) complexes of macrocycles L1 and L3, metal ions are linked by only two μ_2_*-*F^–^ bridges.

**Figure 9:**
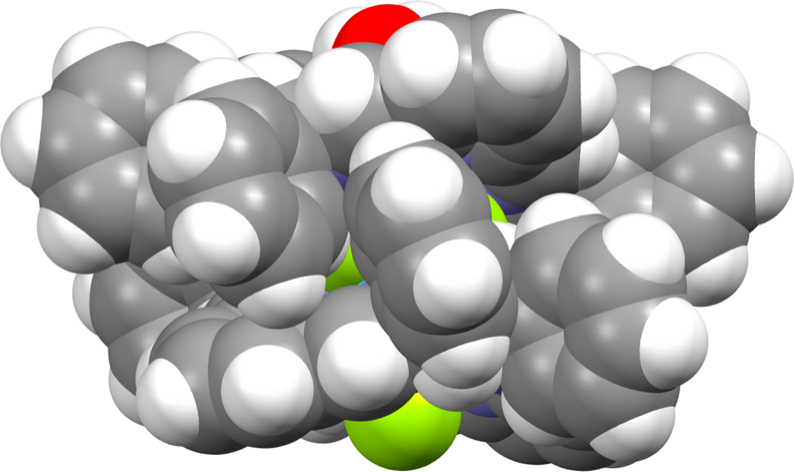
Side view of the complex cation [La_2_(L2^*R*^)_2_(μ_2_-F)_3_F(H_2_O)]^2+^ in space-fill representation showing proximity of phenyl and pyridine rings belonging to two macrocyclic units.

While dinuclear molecular Ln(III) complexes of the di-μ_2_-fluorido type are known,^88−91^ to the best of our knowledge this is a first example of a molecular tri-μ_2_-fluorido dinuclear Ln(III) complex. The Ln_2_(μ_2_-F)_3_ structural motif can be found, however, in the Ln(III)-containing cluster, oligomeric or polymeric compounds. The La–La distance in the complex cation [La_2_(L2^*R*^)_2_(μ_2_-F)_3_F(H_2_O)]^2+^ is 3.71 Å, which is similar value to the values of the corresponding distances 3.66, 3.71, and 3.66 Å observed for the Lu(III), Y(III), and Yb(III) dinuclear complexes of L1 and L3, discussed above. The two macrocyclic units in this complex are close to each other, and phenyl rings at the periphery of macrocycle L2 are rotated in such a way that minimizes steric interactions (Figure 9). In this dimeric complex the macrocyclic units are bent away from the bridging fluorides. In contrast, in the discussed above fluoride-bridged dimers containing macrocycles L1 or L3, the macrocyclic units are twisted but practically not bent.

### NMR Spectra of Fluoride-Bridged Homodinuclear Ln(III) Complexes of Macrocycles L1–L3

**Figure 10:**
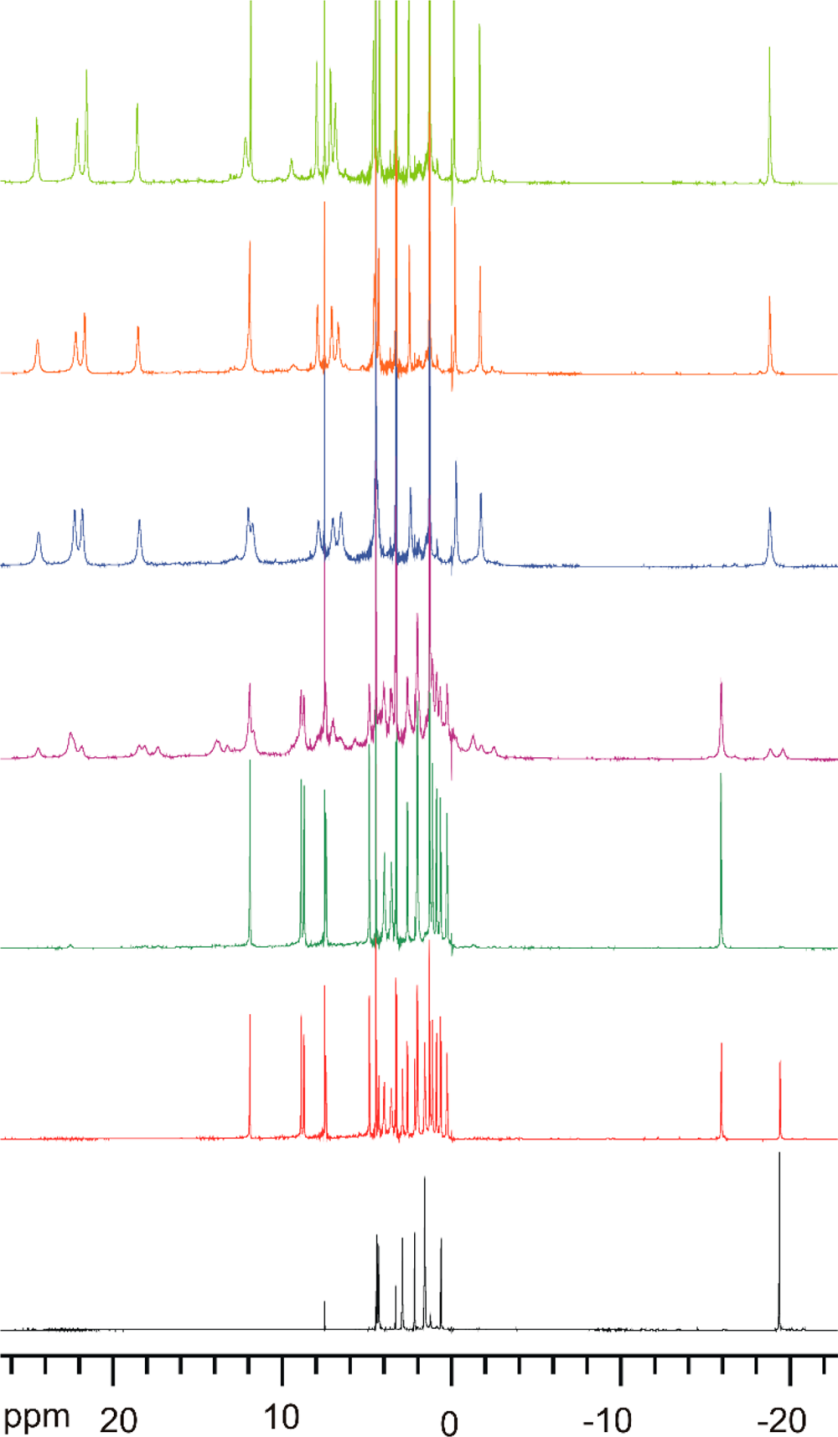
^1^H NMR spectra (CDCl_3_/CD_3_OD 2:1 v/v, 300 K) of the [Eu(L1^*R*^)(NO_3_)_2_](NO_3_) complex and the μ-fluoride dimers generated after addition of increasing amounts of NEt_4_F. From bottom to top: 0, 0.7, 1, 1.5, 2, 3, and 4 equiv of NEt_4_F.

The NMR spectra of paramagnetic macrocyclic Ln(III) complexes may be dramatically changed upon exchange of anions bound in axial positions,^92,93^ including fluoride.^68,69,72^ The number of ^1^H NMR signals observed for the dimeric [Ln_2_(L1)_2_(μ_2_-F)_2_(NO_3_)_2_](NO_3_)_2_ complexes (15 signals, Figure 10 and Figure S8) is increased in comparison with that observed for the starting nitrate derivatives [Ln(L1)(NO_3_)_2_](NO_3_) (eight signals). This observation, points to the switch from the effective *D*_2_ symmetry of the macrocycle in the starting complexes to the *C*_2_ symmetry in the dimer, in accord with the X-ray crystal structures. The lower symmetry of the macrocycle is also confirmed by correlation pattern in the COSY spectrum of the dinuclear Eu(III) complex (Figure S8). Moreover, the range of chemical shifts is greatly changed, particularly for the Yb(III) complexes, where the ^1^H NMR spectrum of the starting mononuclear complex [Yb(L1)(NO_3_)_2_](NO_3_) spans the range of 1 to 26 ppm, while that of the dimeric fluoride derivative spans the range of −49 to 89 ppm (Figure S9). For macrocyclic Yb(III) complexes this kind of profound change of spectral pattern does not arise primarily from the different conformations of the ligand. Instead, it reflects the change of the dominant dipolar (pseudocontact) contribution^94−99^ to the paramagnetic shift caused by the exchange of axial ligands.^92,93^ This effect, in turn, arises from the change of the parameters of the magnetic susceptibility tensor accompanying the change of crystal field at the paramagnetic lanthanide(III) center caused by the replacement of the nitrate or chloride axial ligands with fluoride anions.

The formation of macrocyclic fluoride-bridged Ln(III) complexes in solution was monitored by using ^1^H NMR titration experiments. Gradual addition of solution of sodium fluoride or tetraethylammonium fluoride to mixed methanol/chloroform solutions of [Ln(L1)(NO_3_)_2_](NO_3_) (Ln = Eu, Dy, Yb, Lu, Y) complexes results in subsequent formation of at least three new forms of macrocyclic complexes (Figure 10, Figures S9 and S10). These experiments indicate exchange of axial nitrate anions for fluoride anions. For instance, in the case of Eu(III) derivative addition of up to 1 equiv of F^–^ results in generation of new complex in slow exchange (on the NMR time scale) with the starting complex (Figure 10). This new spectrum is identical with that of the synthesized [Eu_2_(L1^*R*^)_2_(μ_2_*-*F)_2_(NO_3_)_2_](NO_3_)_2_·2H_2_O complex. The addition of more equivalents of fluoride salts brings about further spectral changes consistent with the generation of at least two new fluorido-bridged macrocyclic complexes, most likely corresponding to complex cations with additional terminal fluoride anions: [Ln_2_(L1^*R*^)_2_(μ_2_-F)_2_F(NO_3_)]^2+^ and [Ln_2_(L1^*R*^)_2_(μ_2_-F)_2_F_2_]^2+^.

Similar results were obtained in ^1^H NMR titration experiments with the [Ln(L1)Cl_3_] series of complexes (Figure S10). In this case various fluorido-bridged dinuclear species may coexist in solution. For instance, addition of 1.3 equiv of NEt_4_F to the mixed methanol/chloroform solution [Yb(L1^*R*^)Cl_3_] results in a mixture of three dinuclear species such as [Yb_2_(L1^*R*^)_2_(μ_2_-F)_2_Cl_2_]^2+^, [Yb_2_(L1^*R*^)_2_(μ_2_-F)_2_FCl]^2+^, and [Yb_2_(L1^*R*^)_2_(μ_2_-F)_2_F_2_]^2+^.

Gradual addition of fluoride anions to the solutions of Ln(III) complexes of macrocycles L2 and L3 based on 1,2-diphenylethylenediamine and ethylenediamine, respectively, also brings about substantial NMR changes. However, the ^1^H NMR lines of the paramagnetic derivatives generated after addition of fluoride to the solutions of [Ln(L2)Cl_3_] or [Ln(L3)Cl_3_] are much broader in comparison with those of analogous complexes of L1, and this effect precluded more detailed analysis. The additional line broadening reflects more flexible systems with faster axial ligand exchange and/or conformational changes.

### Formation of Fluoride-Bridged Heterodinuclear Complexes and Self-Sorting of Macrocyclic Units in Solution

**Figure 11:**
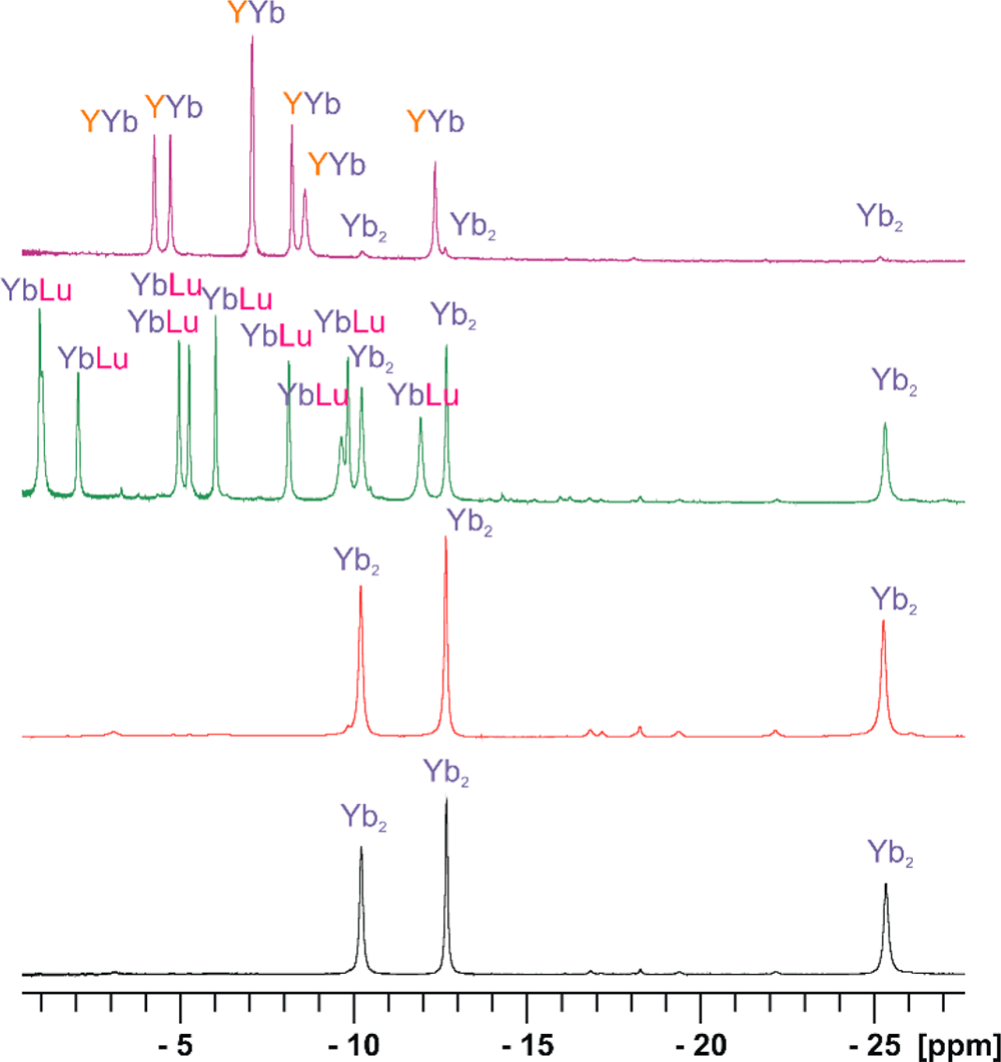
Region of the ^1^H NMR spectra (CDCl_3_/CD_3_OD 2:1 v/v, 300 K) of the μ-fluoride dimers generated by the addition of 0.5 equiv of NEt_4_F to the solutions of [Yb(L1^*S*^)(NO_3_)_2_](NO_3_) complex (black), mixture of [Yb(L1^*S*^)(NO_3_)_2_](NO_3_) and [Lu(L1^*R*^)(NO_3_)_2_](NO_3_) complexes (red), mixture of [Yb(L1^*S*^)(NO_3_)_2_](NO_3_) and [Lu(L1^*S*^)(NO_3_)_2_](NO_3_) complexes (green), or to the mixture of [Yb(L1^*S*^)(NO_3_)_2_](NO_3_) and [Y(L3)(NO_3_)_2_](NO_3_) complexes (violet). Label Yb_2_ indicates the signals of the [Yb_2_(L1^*S*^)_2_(μ_2_-F)_2_(NO_3_)_2_]^2+^ complex, label YbLu denotes signals of the [YbLu(L1^*S*^)_2_(μ_2_-F)_2_(NO_3_)_2_^2+^] complex, and label YYb denotes signals of the [Yb(L1^*S*^)Y(L3)(μ_2_-F)_2_(NO_3_)_2_]^2+^ complex.

**Scheme 1:**
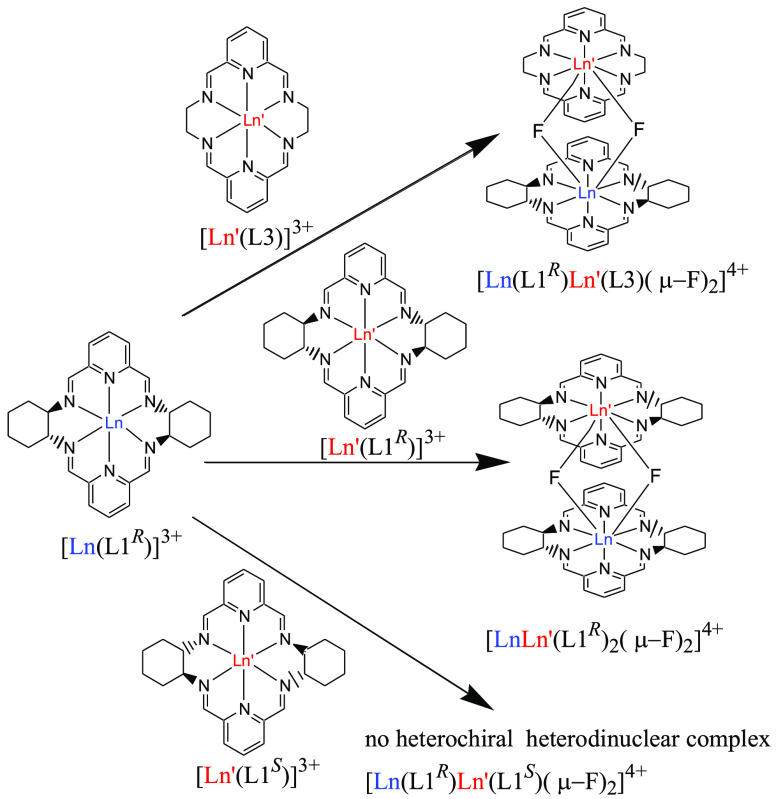


The dinuclear nature of the fluoride derivatives of macrocyclic Ln(III) complexes present in solution may be verified by using two different metal ions. In the ^1^H NMR titration experiments fluoride anions have been added to a mixture of two different mononuclear macrocyclic complexes containing ions Ln(III) and Ln′(III). If the dimeric complexes are formed in these solutions, one should observe, apart from the signals of the homodinuclear complexes, additional set of signals corresponding to the heterodinuclear fluoride-bridged complexes. In particular, these new species should be easily discerned in the case of paramagnetic macrocyclic Ln(III) complexes because of the high sensitivity of chemical shifts of these complexes to any structural modifications. In the case of starting mixture of mononuclear complexes of macrocycle L1 of the same chirality, e.g., a mixture of [Yb(L1^*S*^)(NO_3_)_2_](NO_3_) and [Lu(L1^*S*^)(NO_3_)_2_](NO_3_), indeed the signals of a mixed dimer such as [YbLu(L1^*S*^)_2_(μ_2_-F)_2_(NO_3_)_2_]^2+^ were observed (Figure 11). In this case the dinuclear Yb–Yb, Yb–Lu, and Lu–Lu fluoride-bridged complexes have been formed roughly in the statistical 1:2:1 ratio. A different result was observed with the initial mixture of complexes of opposite chirality, e.g., [Yb(L1^*S*^)(NO_3_)_2_](NO_3_) and [Lu(L1^*R*^)(NO_3_)_2_](NO_3_) (Figure 11). In this case no heterodinuclear Yb–Lu complex was observed. This result is a proof of enantiomeric self-recognition, i.e., narcissistic self-sorting with respect to helicity of macrocyclic units (Scheme 1). This chiral recognition process was not observed in analogous experiments with complexes of macrocycle L2. In this case new heterodinuclear species were observed for the La–Nd couple irrespective of chirality of the macrocycle. With the mixed couples of complexes containing heavier Ln(III) ion or Y(III) the titration experiments suggest that mainly mononuclear fluoride species are generated because heterodinuclear species were not observed.

**Scheme 2:**
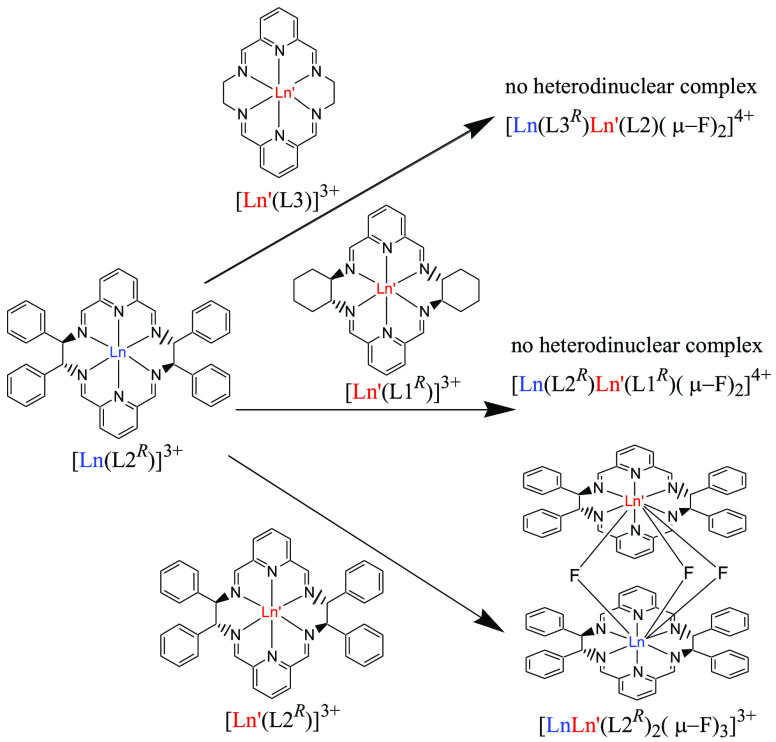


In another set of NMR titration experiments mixtures of two mononuclear complexes containing not only two different metal ions but also two different macrocycles were used. In the case of mixtures of complexes [Ln(L1)Cl_3_] and [Ln′(L2)Cl_3_] after addition of fluoride no dinuclear Ln–Ln′ species containing two different macrocycles L1 and L2 were observed in ^1^H NMR spectra irrespective of the chirality of the macrocycles. This result points to narcissistic self-sorting of macrocyclic units L1 and L2 (Scheme 1). Similar results were obtained for the starting mononuclear complexes [Ln(L2)Cl_3_] and [Ln′(L3)Cl_3_] pointing to narcissistic self-sorting of macrocyclic units L2 and L3. In contrast, sociable self-sorting of macrocycles L1 and L3 was observed during formation of heterodinuclear fluoride-bridged complexes (Scheme 2). For instance, addition of tetraethylammonium fluoride to the solution of the mixture of [Yb(L1^*S*^)Cl_3_] and [Y(L3)Cl_3_] resulted in formation of the heterodinuclear [Yb(L1^*S*^)Y(L3)(μ_2_-F)_2_Cl_2_]^2+^ complex. Moreover, in the reaction of NEt_4_F with a mixture of analogous nitrate derivatives [Yb(L1^*S*^)(NO_3_)_2_](NO_3_) and [Y(L3)(NO_3_)_2_](NO_3_) the initial products contain the mixed Yb/L1-Y/L3 fluorido-bridged complex [Yb(L1^S^)Y(L3)(μ_2_-F)_2_(NO_3_)_2_]^2+^ and only traces of the homodinuclear [Yb_2_L1^*S*^_2_(μ_2_-F)_2_(NO_3_)_2_]^2+^ complex (Figure 11). This selectivity was not observed for the L3 complex of larger Eu(III) ion where the mixed [Y(L1^*S*^)Eu(L3)(μ_2_-F)_2_(NO_3_)_2_]^2+^ complex was formed together with homodinuclear complexes in a ratio close to the statistical distribution. Similarly, our attempts to isolate the heterodinuclear complex containing two different macrocyclic units as pure solids were so far unsuccessful, and the crystallization process seems to shift the equilibrium between various dinuclear forms. It should be mentioned that the heteronuclear mixed-lanthanide complexes characterized in solution are rare.^100−117^

### Chirality Transfer in Heterodinuclear [Yb(L1)Y(L3)(μ_2_-F)_2_(NO_3_)_2_]^2+^ Complexes

Because the macrocycle L3 is based on ethylenediamine lateral chains and is achiral, both its mononuclear complexes [Ln(L3)(NO_3_)_2_](NO_3_) and dinuclear complexes [Ln_2_L3_2_(μ_2_*-*F)_2_(NO_3_)_2_](NO_3_)_2_ do not exhibit CD signals. Nevertheless, the X-ray crystal structures indicate that this macrocycle adopts chiral, helical conformation in its monomeric Ln(III) complexes^118^ as well as in the dimeric fluoride-bridged complex discussed above. Moreover, both macrocyclic L3 units are of the same chirality within the dimer. Because this homohelical arrangement is analogous to the homochiral formation of dinuclear complexes of L1, it is likely that in the mixed [Ln(L1)Ln′(L3)(μ_2_-F)_2_(NO_3_)_2_]^2+^ complex cation the macrocycle L3 has to assume the same direction of helical twist as macrocycle L1 to minimize steric interactions between the two macrocyclic units. The Λ and Δ conformations are equally probable in the dinuclear [Ln_2_(L3)_2_(μ_2_*-*F)_2_(NO_3_)_2_](NO_3_)_2_ complexes of L3. On the other hand, the macrocyclic unit L3 may adopt preferable Δ conformation when it is in close contact with the Δ/L1^*S*^ macrocyclic unit in the mixed dimer [Ln(L1^S^)Ln′(L3)(μ_2_-F)_2_(NO_3_)_2_]^2+^. Conversely, the preferable Λ conformation of L3 may be present in the [Ln(L1^R^)Ln′(L3)(μ_2_-F)_2_(NO_3_)_2_]^2+^ complex cations. CPL and CD spectra indicate that this kind of chirality transfer from the chiral macrocycle L1 to achiral macrocycle L2 is indeed happening.

**Figure 12:**
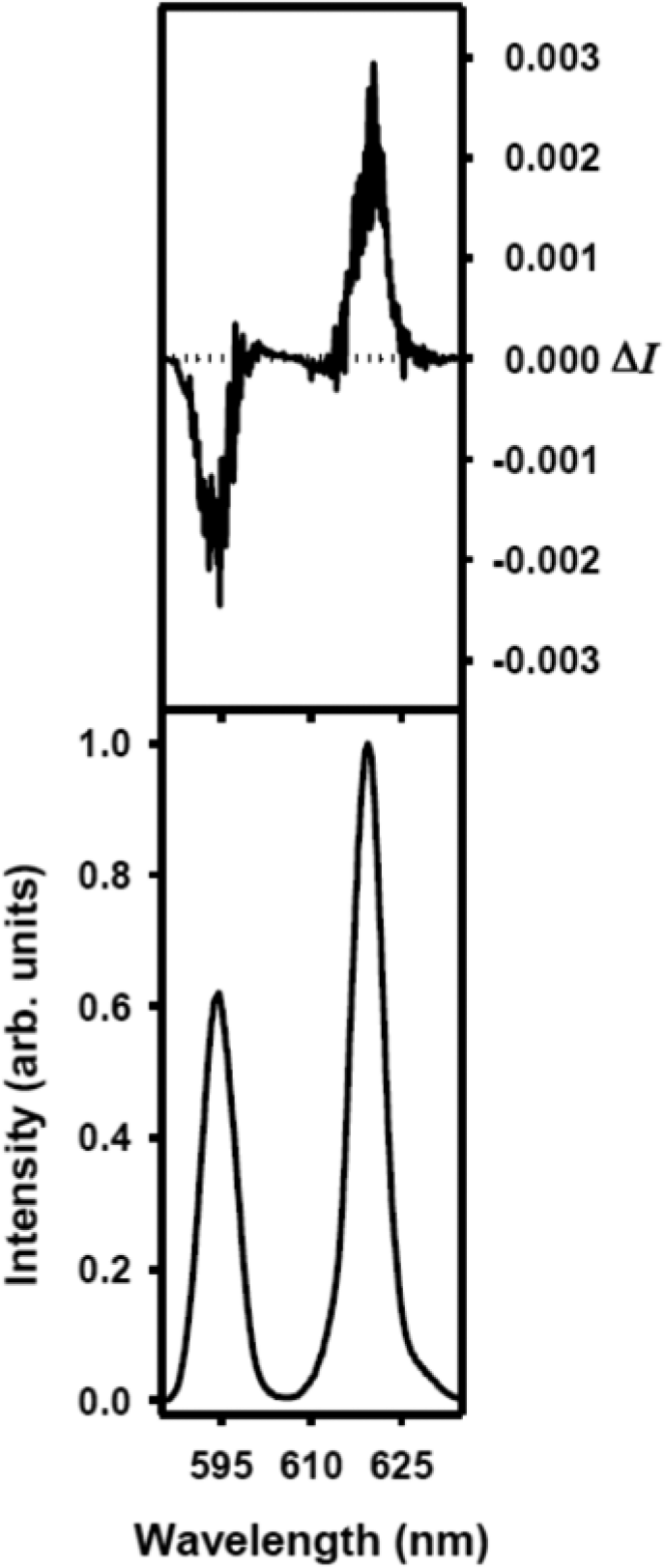
CPL (upper curves) and total luminescence (lower curves) spectra for the ^5^D_0_ → ^7^F_1_ and ^5^D_0_ → ^7^F_2_ transitions of the mixture of [Y_2_(L1^*S*^)_2_(μ_2_-F)_2_(NO_3_)_2_](NO_3_)_2_, [Eu_2_(L3)_2_(μ_2_-F)_2_(NO_3_)_2_](NO_3_)_2_, and [Y(L1^*S*^)Eu(L3)(μ_2_-F)_2_(NO_3_)_2_](NO_3_)_2_ complexes in 2 mM deuterated 2:1 chloroform:methanol at 295 K, upon excitation at 321 nm.

**Scheme 3:**
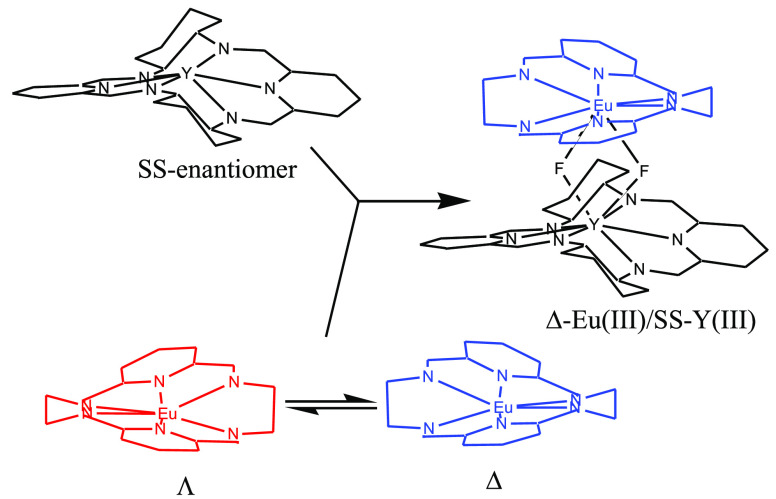


Thus, the CPL signal was monitored for the mixture of [Y_2_(L1^*S*^)_2_(μ_2_-F)_2_(NO_3_)_2_](NO_3_)_2_, [Eu_2_(L3)_2_(μ_2_-F)_2_(NO_3_)_2_](NO_3_)_2_, and [Y(L1^*S*^)Eu(L3)(μ_2_-F)_2_(NO_3_)_2_](NO_3_)_2_ complexes generated from the mixture of [EuL3)(NO_3_)_2_](NO_3_) and [Y(L1^*S*^)(NO_3_)_2_](NO_3_) (Figure 12). The transition that was measured for this sample dissolved in deuterated 2:1 chloroform/methanol solution was the magnetic dipole allowed transition ^5^D_0_ → ^7^F_1_ for Eu(III). In addition, we also recorded the CPL activity for the (Eu) ^5^D_0_ → ^7^F_2_. The *g*_lum_ values are −0.05 and +0.04 for the (Eu) ^5^D_0_ → ^7^F_1_ and ^5^D_0_ → ^7^F_2_ transitions. The observation of an Eu(III)-centered CPL activity clearly indicates that the Eu(III) resides in a chiral nonracemic complex. The signals cannot arise from the enantiopure component [Y_2_(L1^*S*^)_2_(μ_2_-F)_2_(NO_3_)_2_](NO_3_)_2_ since it is nonluminescent and CPL silent. They also cannot arise from the Eu_2_(L3)_2_(μ_2_-F)_2_(NO_3_)_2_](NO_3_)_2_ component since, here, Eu(III) resides in a racemic mixture of Δ and Λ conformations of the achiral ligand L3. The conclusion is that CPL activity has to arise from the [Y(L1^*S*^)Eu(L3)(μ_2_-F)_2_(NO_3_)_2_](NO_3_)_2_ component of the mixture and that the Eu(III) ion in the heterodinuclear complex cation [Y(L1^*S*^)Eu(L3)(μ_2_-F)_2_(NO_3_)_2_]^2+^ is bound by achiral L3 which has assumed only one of the two possible directions of the helical twist (Scheme 3). It should be mentioned that the NMR spectra of the sample used for CPL measurements do not indicate characteristic paramagnetic signals of species where the Eu(III) ion is coordinated to the chiral macrocycle. Thus, the observed CPL signal of Eu(III) does not arise from the metal ion dissociation and scrambling between the L1 and L3 sites.

**Figure 13:**
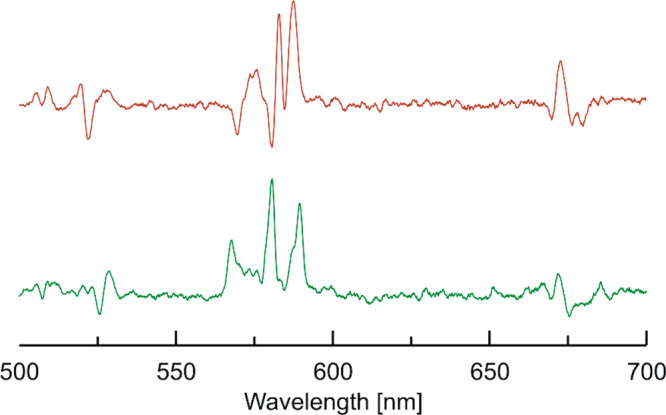
CD spectrum of CDCl_3_/CD_3_OD 2:1 v/v solution of the equimolar mixture of [Y(L1^*S*^)(NO_3_)_2_](NO_3_) and [Nd(L3)(NO_3_)_2_](NO_3_) complexes generated after addition of 1 equiv (top) and 2 equiv (bottom) of NEt_4_F.

Similarly, chirality transfer from the enantiopure macrocycle L1 to the achiral macrocycle L3 is confirmed by the CD spectra. As expected, the CDCl_3_/CD_3_OD 2:1 v/v solution of the mixture of the monomeric complexes [Nd(L3)(NO_3_)_2_](NO_3_) and [Y(L1^*S*^)(NO_3_)_2_](NO_3_) complexes does not generate CD signal in visible light range. Conversely, the [Y(L1*^S^*)Nd(L3)(μ_2_-F)_2_(NO_3_)_2_]^2+^ complex generated from this mixture by addition of 1 equiv of NEt_4_F gives rise to weak and narrow CD signals in the 500–700 nm region (Figure 13). This kind of signal cannot arise from the macrocyclic units, which do not absorb in this region. Similarly, it does not arise from the Y(III) ion which lacks f–f transitions. Thus, the observed CD signals have to arise from the f–f transitions of the Nd(III) ions, and they reflect the nonracemic, chiral environment of these ions bound by the L3 macrocycle. This chiral environment of Nd(III) is due to generation of preferred direction of helical twist of the achiral macrocycle L3 caused by the steric interactions with the macrocycle L1 within the heterodinuclear complex. NMR data were used to verify that the observed CD signals due to f–f transitions did not arise from metal ion exchange between the L1 and L2 macrocycles. Further addition of fluoride anions results in the change of CD signals. These changes reflect variations of crystal field parameters of Ln(III) ion and the formation of new species (most likely with additional terminal fluorides bound to Nd(III) centers), in accord with the results of NMR titrations. We have recently observed similar CD effects in the hydroxo-bridged dinuclear species.^21^

**Figure 14:**
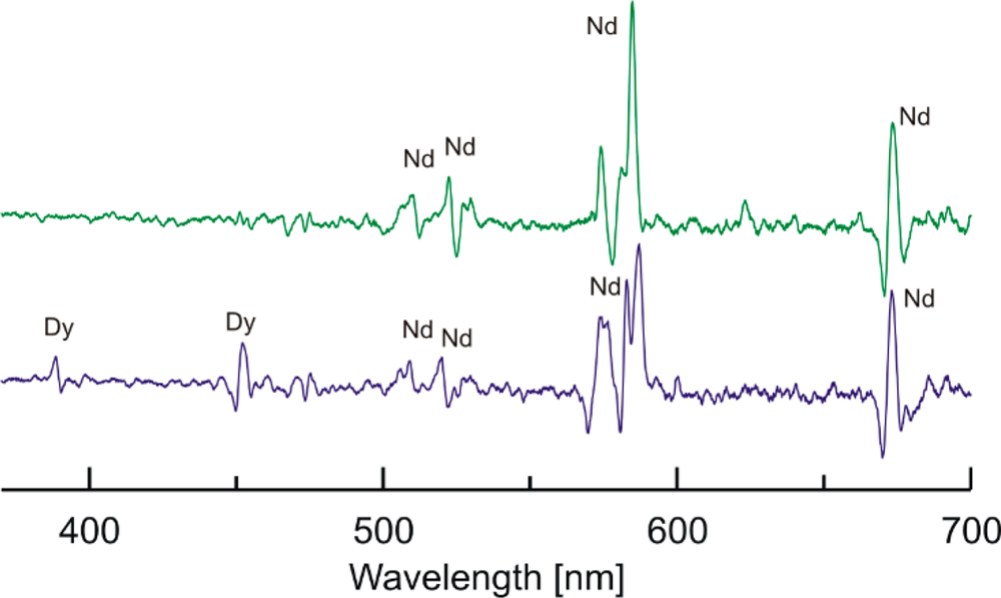
Top: CD spectrum of water solution of the equimolar mixture of [Nd(L1^*S*^)(NO_3_)_2_](NO_3_) and [Dy(L3)(NO_3_)_2_](NO_3_) complexes. Bottom: CD spectrum of the same mixture after addition of 1 equiv of NEt_4_F. Label Dy denotes f–f transitions of the dysprosium(III) cation bound by the achiral L3 macrocycle, and the label Nd denotes f–f transitions of neodymium(III) bound by the chiral L1 macrocycle.

In another CD experiment a starting mixture of monomeric [Nd(L1)(NO_3_)_2_](NO_3_) and [Dy(L3)(NO_3_)_2_](NO_3_) was used. This mixture generated CD signals of the Nd(III) ion residing within the chiral macrocycle L1, but no CD signals due to Dy(III) ions residing within achiral macrocycle L3 were observed. After addition of 1 equiv of NEt_4_F, the Nd(III) signal changed due to the formation of fluoride-bridged dinuclear species, and a new weak signal corresponding to the f–f transitions of Dy(III) ion appeared due to chirality transfer (Figure 14). It is worth mentioning that the Nd(III) signals of the generated dinuclear species are very similar to those generated after addition of 1 equiv of fluoride in the previous experiment described above. Thus, the shape of the Nd(III) CD signal is analogous for the [Nd(L1^*S*^)Dy(L3)(μ_2_-F)_2_(NO_3_)_2_]^2+^ and [Y(L1^S^)Nd(L3)(μ_2_-F)_2_(NO_3_)_2_]^2+^ dinuclear species. This confirms that the macrocycles L1 and L3 assume the same direction of helical twist in these two different fluoride-bridged complexes. The similarity of these CD signals also indicates that the kind of hexaaza-macrocycle does not influence much the crystal field parameters of the Nd(III).

## Conclusions

Ln(III) complexes of hexaaza-macrocycles L1–L3 tend to form dimers where two macrocyclic units are linked by two or three bridging fluoride anions. This contrasts the behavior of Ln(III) complexes with cyclen-based tetraaza-marocycles where dimers are linked by single fluoride anions or mononuclear complexes with terminal fluoride are generated in reactions with fluoride salts. This difference reflects more open axial coordination spheres of the complexes of hexaaza-macrocycles. Within these dimeric complexes the macrocyclic units L1–L3 are in a relatively close contact, and the steric interactions between them leads to sorting phenomena. Formation of the dinuclear fluoride-bridged complexes based on the chiral macrocycle L1 is accompanied by narcissistic sorting of macrocyclic units based on chirality (enantiomeric self-recognition). In the systems containing the mixture of Ln(III) complexes of two different macrocyclic ligands the formation of fluoride derivatives is influenced by matching of the shapes of the two macrocyclic units. Thus, the reactions of a mixture of complexes of macrocycles L1 and L2 or macrocycles L2 and L3 is accompanied by narcissistic sorting of macrocyclic units. On the other hand, in the case of the pair of macrocyclic complexes L1 and L3, the formation of fluoride-bridged dinuclear complexes is accompanied by social sorting of macrocyclic units. In this case the formation of mixed dimers is accompanied by chirality transfer from the chiral macrocycle L1 to achiral macrocycle L3. Steric interactions between macrocyclic units in heterodinuclear complexes of the type [Ln(L1)Ln′(L3)(μ_2_-F)_2_(NO_3_)_2_]^2+^ cause the macrocycle L3 to adopt a preferred direction of helical twist matching the helicity of the L1 unit. In turn, the direction of helical twist of macrocycle L1 is predetermined by the configuration at the chiral carbon atoms. This chirality transfer effect manifests itself in the appearance of CPL and CD signals corresponding to f–f transitions of Ln(III) ions bound by the achiral L3 unit.

## Experimental Section

Details of structure determination and CPL measurements are provided in the Supporting Information.

### Synthesis of Mononuclear Complexes

The Ln(III) complexes of macrocycles L1 and L3 have been obtained as reported previously.^20,21,118^ The [Ln(L2^*R*^)Cl_3_] (Ln = La, Ce, Eu, Dy) complexes were obtained in a similar manner as reported previously,^85,86^ as were new complexes of this type (Ln = Pr, Nd, Tb, and Y) as well as enantiomeric complexes [Ln(L2)^*S*^Cl_3_]. In a typical procedure 2,6-diformylpyridine (135 mg, 1 mmol), (1*R*,2*R*)-1,2-diphenylethylenediamine (212 mg, 1 mmol), and the appropriate lanthanide(III) chloride hexahydrate (0.5 mmol) were refluxed in 20 mL of methanol for 3 h. After cooling, the volume was reduced to ca. 3 mL by using a rotary evaporator. The white product that formed was filtered, washed with methanol, and dried in a vacuum.

[Pr(L2^*R*^)Cl_3_]·2H_2_O. Anal. Calcd for C_42_H_38_Cl_3_N_6_O_2_Pr: C, 55.68; H, 4.23; N, 9.28. Found C, 55.45; H, 4.33; N, 9.25. ^1^H NMR (CDCl_3_/CD_3_OD 2:1 v/v, 298 K, 500 MHz): δ_H_ 22.06, 14.47, 13.59, 7.35, 7.28, 6.34, −1.74. IR (KBr pellet, cm^–1^): 3351, 3052, 1650, 1591, 1496, 1455, 1271, 1163, 1036, 1011, 766, 705, 581.

[Nd(L2^*R*^)Cl_3_]·4H_2_O. Anal. Calcd for C_42_H_42_Cl_3_N_6_NdO_4_: C, 53.36; H, 4.48; N, 8.89. Found C, 53.53; H, 4.84; N, 8.91. ^1^H NMR (CDCl_3_/CD_3_OD 2:1 v/v, 298 K, 500 MHz): δ_H_ 24.59, 12.51, 11.64, 7.66, 7.61, 5.24. IR (KBr pellet, cm^–1^): 3401, 3053, 1650, 1591, 1494, 1464, 1453, 1273, 1168, 1038, 1013, 767, 706, 584.

[Tb(L2^*R*^)Cl_3_]·3H_2_O. Anal. Calcd for C_42_H_40_Cl_3_N_6_O_3_Tb: C, 53.55; H, 4.28; N, 8.92. Found C, 53.24; H, 4.45; N, 8.83. ^1^H NMR (CDCl_3_/CD_3_OD 2:1 v/v, 298 K, 500 MHz): δ_H_ 60.0, 52.45, 7.7, 1.13, −17.4. IR (KBr pellet, cm^–1^): 3401, 3056, 1649, 1636, 1594, 1494, 1469, 1454, 1279, 1169, 1044, 1014, 766, 711, 700, 586.

[Y(L2^*R*^)Cl_3_]·3H_2_O. Anal. Calcd for C_42_H_40_Cl_3_N_6_O_3_Y: C, 57.85; H, 4.62; N, 9.64. Found C, 57.47; H, 4.98; N, 9.52. ^1^H NMR (CDCl_3_/CD_3_OD 2:1 v/v, 298 K, 500 MHz): δ_H_ 8.16 (s, 4H), 8.14 (t, 2H, *J* = 7.7 Hz), 7,72 (d, 4H, *J* = 7.7 Hz), 7.73–7.25 (m, 20H), 5.82 (s, 4H). ^13^C{^1^H} NMR (CDCl_3_/CD_3_OD 2:1 v/v, 298 K, 126 MHz): δ 164.03, 151.39, 142.18, 134.91, 130.39, 129.94, 129.77, 129.58, 74.21. IR (KBr pellet, cm^–1^): 3370, 3056, 1650, 1638, 1594, 1494, 1469, 1454, 1279, 1169, 1044, 1014, 766, 700, 586.

### Synthesis of Dinuclear Complexes

[Nd_2_(L1^*S*^)_2_(μ_2_-F)_2_(NO_3_)_2_](NO_3_)_2_·2H_2_O. 75.7 mg (0.1 mmol) of [Nd(L1^*S*^)(NO_3_)_2_](NO_3_) was suspended in 2 mL of methanol and mixed with the solution of 5.8 mg of KF (0.1 mmol) in 100 μL of methanol. The mixture was vigorously stirred for 2 h, filtered, and washed with methanol. Yield 43 mg, 59%. Anal. Calcd for C_52_H_64_F_2_N_16_Nd_2_O_14_: C, 42.67; H, 4.41; N, 15.31. Found: C, 42.29; H, 4.02; N, 15.39. ^1^H NMR (CDCl_3_/CD_3_OD 2:1 v/v, 298 K, 500 MHz): δ_H_ 23.38, 13.48, 10.97, 8.66, 6.60, 3.75, 3.50, 2.91, 2.47, 1.86, 1.00, 0.83, −1.50, −1.71. IR (KBr pellet, cm^–1^): 3436, 3069, 2931, 2863, 1649, 1590, 1494, 1465, 1451, 1384, 1354, 1309, 1171, 1100, 1042, 1010, 824, 580.

[Eu_2_(L1^*R*^)_2_(μ_2_-F)_2_(NO_3_)_2_](NO_3_)_2_·2H_2_O. 76.5 mg (0.1 mmol) of [Eu(L1^*R*^)(NO_3_)_2_](NO_3_) was suspended in 3 mL of methanol and mixed with the solution of 5.8 mg of KF (0.1 mmol) in 100 μL of methanol. The mixture was vigorously stirred for 48 h, and the precipitate was filtered, washed with 1 mL of methanol, and dried in air. Yield 48 mg, 65%. Anal. Calcd for C_52_H_64_Eu_2_F_2_N_16_O_14_: C, 42.23; H, 4.36; N, 15.15. Found: C, 42.20; H, 4.04; N, 15.06. NMR (CDCl_3_/CD_3_OD 2:1 v/v, 298 K, 500 MHz): δ_H_ 11.93, 8.89, 8.76, 7.44, 4.86, 3.94. 3.62, 2.62, 2.02, 1.13, 0.89, 0.67, 0.27, −15.86. IR (KBr pellet, cm^–1^): 3435, 3070, 2931, 2863, 1652, 1592, 1494, 1466, 1451, 1384, 1354, 1312, 1171, 1102, 1045, 1009, 823, 580.

[Dy_2_(L1^*R*^)_2_(μ_2_-F)_2_(NO_3_)_2_](NO_3_)_2_·3H_2_O. 77.5 mg (0.1 mmol) of [Dy(L1^*R*^)(NO_3_)_2_](NO_3_) was dissolved in 3 mL of the 2:1 v/v mixture of chloroform/methanol and combined with the solution of 16.7 mg (0.1 mmol) of NEt_4_F·H_2_O in 100 μL of methanol. The clear solution was left to slowly evaporate in air for 2 days, and the volume was reduced to ca. 1 mL. The formed crystalline deposit was filtered, washed with 1 mL of methanol, and dried in air. Yield 37 mg 49%. Anal. Calcd for C_52_H_66_Dy_2_F_2_N_16_O_15_: C, 41.14; H, 4.38; N, 14.76. Found: C, 41.37; H, 4.48; N, 14.74. NMR (CDCl_3_/CD_3_OD 2:1 v/v, 298 K, 500 MHz): δ_H_ 27.56, −9.47, −11.24, −18.77, −24.26, −57.27, −60.17, −63.27, −141.71, −152.00, −172.32. IR (KBr pellet, cm^–1^): 3435, 3072, 2931, 2861, 1653, 1593, 1494, 1464, 1384, 1357, 1315, 1166, 1103, 1047, 1009, 817, 581.

[Y_2_(L3)_2_(μ_2_-F)_2_(NO_3_)_2_](NO_3_)_2_·H_2_O. A mixture of 61.1 mg (0.1 mmol) of [Y(L3)(NO_3_)_2_](NO_3_) and 16.7 mg (0.1 mmol) of NEt_4_F·H_2_O in 5 mL of the 2:1 v/v mixture of chloroform/methanol was stirred for 1 h and left to stand for 24 h. The suspension was filtered, and the precipitate washed with 1 mL of methanol and dried in air. Yield 39 mg, 70%. Anal. Calcd for C_36_H_38_F_2_N_16_O_13_Y_2_: C, 38.65; H, 3.42; N, 20.03. Found: C, 38.88; H, 3.63; N, 19.63. ^1^H NMR (CDCl_3_/CD_3_OD 2:1 v/v, 298 K, 500 MHz): δ_H_ 8.46 (s, 4H, 8.32 (t, 2H, *J* = 7.6 Hz), 8.02 (d, 4H, *J* = 7.6 Hz), 3.41 (s, 4H, br), 3.04 (s, 4H, br). ^13^C{^1^H} NMR (CDCl_3_/CD_3_OD 2:1 v/v, 298 K, 126 MHz): δ 163.25, 151.45, 142.33, 129.15, 60.2. IR (KBr pellet, cm^–1^): 3435, 3075, 2922, 2862, 1663, 1594, 1467, 1384, 1357, 1304, 1162, 1035, 1009, 817, 760, 667, 649, 602.

## Supporting Information Available

Crystallographic data and details of structure refinement; details of CPL measurements; Figures S1–S10 (molecular structures and NMR spectra) (PDF)

## Accession Codes

CCDC 2112764–2112769 contain the supplementary crystallographic data for this paper. These data can be obtained free of charge via www.ccdc.cam.ac.uk/data_request/cif, or by emailing data_request@ccdc.cam.ac.uk, or by contacting The Cambridge Crystallographic Data Centre, 12 Union Road, Cambridge CB2 1EZ, UK; fax: +44 1223 336033.
