# Supplementary material for: Sorting Phenomena and Chirality Transfer in Fluoride-Bridged Macrocyclic Rare Earth Complexes
Source: Inorg Chem. 2021 Nov 16;60(23):18442–54. doi: 10.1021/acs.inorgchem.1c03034 (PMC8653217; doi:10.1021/acs.inorgchem.1c03034)
Supplement: Supplementary file 1 — ic1c03034_si_001.pdf [file ic1c03034_si_001.pdf]

Supporting Infromation

Sorting Phenomena and Chirality Transfer in Fluoride-Bridged Macrocyclic Rare Earth Complexes

Katarzyna Ślepokura[†], *Trevor A. Cabreros*[‡], *Gilles Muller*[‡]* *and Jerzy Lisowski*[†]*

[†]Department of Chemistry, University of Wrocław, 14 F. Joliot-Curie, 50-383 Wrocław, Poland. Fax: 48 71 3282348; Tel: 48 71 3757252

E-mail: jerzy.lisowski@chem.uni.wroc.pl

[‡]Department of Chemistry, San José State University, One Washington Square, San José, CA 95192-0101, USA. Fax: 408-924-4945, Tel: 408-924-2632

E-mail: gilles.muller@sjsu.edu

Measurements

The NMR spectra were taken on Bruker Avance 500 and Bruker Avance III 600 MHz spectrometers. The elemental analyses were carried out on a Perkin-Elmer 2400 CHN elemental analyzer. The CD spectra were recorded on Jasco J-1500 CD spectrometer. IR spectra were recorded with Bruker Vertex 70 FTIR instrument, using KBr pellets. Circularly polarized luminescence (CPL) and total luminescence spectra were recorded on an instrumentation described previously.[1] In short, the instrumentation is equipped with a 1000 W xenon arc lamp from a Spex FluoroLog-2 spectrofluorometer, with excitation and emission monochromators of dispersions 4 nm/mm (SPEX, 1681B). The optical detection system consisted of a focusing lens long-pass filter and 0.22 m monochromator. A cooled EMI-9558B photomultiplier tube operating in photon-counting mode detected the emitted light. The standard deviation, σ_d, in the measurement of the luminescence dissymmetry factor, g_{lum}, is defined as $\sigma_d = (2 / N)^{1/2}$ where N is the total number of photon-pulses counted. One can see that the determination of accurate g_{lum} values can be done in a short time for transitions associated with large g_{lum} values of highly luminescent compounds, whereas a longer time of

collection is required for transitions associated with small g_{lum} values of weakly luminescent systems for achieving the same percent error. As the time required for measuring a CPL spectrum is dependent on the intensity of the luminescence of the system of interest and the “chirality” of the transition analyzed, the photo-pulses are collected for the same amount of time at each wavelength. As a result, the relative error at each of these wavelengths is the same in the CPL spectrum measured. The g_{lum} values are given with a standard deviation, σ_d, of ±0.01. It should be noted that a value of 0 for g_{lum} corresponds to no circular polarization, while the absolute maximum value is 2. It is often the case that rigid Ln(III) systems exhibit large g_{lum} values, while racemic or other types of mixtures give g_{lum} values in the range of ~10^{-2}-10^{-3}.[2] All measurements were performed in a quartz cuvette with a path length of 1.0 cm.

Crystal structure determinations

Single crystals of $[La(L2^R)Cl_3]\cdot 2.5MeOH\cdot 0.5H_2O$ and $\{[Tb(L2^R)Cl_2(MeOH)][Tb(L2^R)Cl_2(H_2O)]\}Cl_2\cdot 9MeOH\cdot H_2O$ were obtained by slow evaporation of methanol solutions. The crystals of dimeric complex $[Y_2(L3)_2(\mu_2\text{-}F)_2(NO_3)_2](NO_3)_2\cdot CHCl_3\cdot MeOH\cdot H_2O$ were obtained by slow evaporation of mixed chloroform/methanol solution. The crystals of $[Lu_2(L1^R)_2(\mu_2\text{-}F)_2(NO_3)_2](NO_3)_2\cdot CHCl_3\cdot 3H_2O$ complex were obtained by slow evaporation of mixed chloroform/methanol solution containing equimolar mixture of $[Lu(L1^R)(NO_3)_2](NO_3)$ and $NEt_4F\cdot H_2O$. The crystals of $[Yb_2(L1^R)_2(\mu_2\text{-}F)_2F(H_2O)]Cl_3\cdot 3.5MeOH\cdot 4.5H_2O$ were obtained by slow evaporation of methanol solution containing 1 equivalent of $[Yb(L1^R)Cl_3]$ and 1.5 equivalents of KF. The crystals of $[La_2(L2^R)_2(\mu_2\text{-}F)_3F(H_2O)]Cl_2\cdot 5MeOH\cdot H_2O$ complex were obtained by slow evaporation of methanol solution containing 1 equivalent of $[La(L2^R)Cl_3]$ and 2 equivalents of $NEt_4F\cdot H_2O$.

Single-crystal X-ray diffraction data were collected at 100 or 120 K, on Kuma KM4-CCD, Oxford Diffraction Xcalibur PX, Agilent Technologies Xcalibur R or Agilent Technologies Gemini Ultra κ-geometry diffractometers using Mo $K\alpha$ or Cu $K\alpha$ radiation (for details see Supporting Table S1). Data collections, cell refinements, data reductions and analyses were carried out with CrysAlisPro.[3] Data were corrected for Lorentz, polarization and absorption effects (analytical or empirical; multi-scan). Crystal structures of $[La(L2^R)Cl_3]\cdot 2.5MeOH\cdot 0.5H_2O$ (which is isomorphous with the cerium(III) compound;[4] atomic coordinates but not the atom-numbering scheme consistent), $\{[Tb(L2^R)Cl_2(MeOH)][Tb(L2^R)Cl_2(H_2O)]\}Cl_2\cdot 9MeOH\cdot H_2O$ (isomorphous with the

dysprosium compound, {[Dy(L2^{R})Cl_2(MeOH)][Dy(L2^{R})Cl_2(H_2O)]}Cl_2·8MeOH;[5] although the atomic coordinates and the atom-numbering scheme are changed), [Lu_2(L1)$_2$(μ_2-F)$_2$(NO_3)$_2$](NO_3)$_2$·$CHCl_3$·3H_2O, [Yb_2(L1^{R})$_2$(μ_2-F)$_2$F(H_2O)]Cl_3·3.5MeOH·4.5H_2O and [La_2(L2^{R})$_2$(μ_2-F)$_3$F(H_2O)]Cl_2·5MeOH·H_2O were solved using a dual-space algorithm with SHELXT-2014 program[6] or by direct methods with the use of SHELXS-2013 program,[7] and refined on F^2 by a full-matrix least-squares method using the SHELXL-2014/7 program.[8] Crystal of [Y_2(L3)$_2$(μ_2-F)$_2$(NO_3)$_2$](NO_3)$_2$·$CHCl_3$·MeOH·H_2O is isomrphous with the crystal of [Y_2(L3)$_2$(μ_2-OH)$_2$(H_2O)$_2$](NO_3)$_4$·4H_2O,[9] therefore the refinement of its structure was started by using the coordinates (along with the labelling scheme) of ordered heavy atoms taken from the crystal of that coordination compound. Finally, the inverted model has been used.

All non-H atoms in the crystal of Tb^{III} compound, and those with site occupation factors (SOFs) > 0.5 (or with SOF = 0.5 for Cl^-) in the crystals of Lu^{III}, Yb^{III}, Y^{III} and both La^{III} compounds were refined with anisotropic atomic displacement parameters.

Two phenyl rings and all bridging F^- anions in [La_2(L2^{R})$_2$(μ_2-F)$_3$F(H_2O)]Cl_2·5MeOH·H_2O were found to be disordered and were refined in two positions each. All of the methanol molecules in [La(L2^{R})Cl_3]·2.5MeOH·0.5H_2O, as well as one of the non-coordinated NO_3^- anions in [Lu_2(L1^{R})$_2$(μ_2-F)$_2$(NO_3)$_2$](NO_3)$_2$·$CHCl_3$·3H_2O, were also refined in two positions each. Two of the MeOH molecules in the crystal of [La(L2^{R})Cl_3]·2.5MeOH·0.5H_2O and most of them in [La_2(L2^{R})$_2$(μ_2-F)$_3$F(H_2O)]Cl_2·5MeOH·H_2O and [Yb_2(L1^{R})$_2$(μ_2-F)$_2$F(H_2O)]Cl_3·3.5MeOH·4.5H_2O were additionally not fully occupied. The same for some water molecules in Yb^{III} crystal. All of the water molecules in the crystals of Lu^{III} compound as well as in Y^{III} derivative, most of them in [La_2(L2^{R})$_2$(μ_2-F)$_3$F(H_2O)]Cl_2·5MeOH·H_2O and one H_2O in Yb^{III} crystal were modeled as disordered in two, three or four positions. For details see the CIF files. Some geometrical restraints (DFIX, FLAT, SAME instructions in the SHELXL-2014), restraints on anisotropic displacement parameters (SIMU, ISOR), constraints on the coordinates and U_{ij} (EXYZ and EADP), and restraints on the sum of SOFs (SUMP) were applied in the refinement procedures, when appropriate.

H atoms were included using geometrical considerations or were found in difference Fourier maps. Some of them (e.g. O-bound) initially were refined freely, but in the final refinement cycles, C-bound H atoms in all structures, as well as those from ligand/solvent OH groups (MeOH, H_2O) were repositioned in their calculated positions and refined using a riding model, with O–H = 0.84 Å and C–H = 0.95–1.00 Å, and with U_{iso}(H) = 1.5U_{eq}(O), 1.2U_{eq}(CH, CH_2) or 1.5U_{eq}(CH_3). Water H atoms in the crystal of Tb^{III} compound were refined with the O–H

bond lengths restrained to 0.840(2) Å, H···H distances restrained to 1.360(2) Å and then a rigid group or riding model constraints were applied (AFIX 6 or AFIX 3 instructions in SHELXL). The same for four water molecules in Yb^{III} crystal and the coordinated H_2O in $La_2(L2^{R})_2(\mu_2\text{-}F)_3F(H_2O)]Cl_2\cdot 5MeOH\cdot H_2O$. Remaining water H atoms in $[Yb_2(L1^{R})_2(\mu_2\text{-}F)_2F(H_2O)]Cl_3\cdot 3.5MeOH\cdot 4.5H_2O$ and $[La_2(L2^{R})_2(\mu_2\text{-}F)_3F(H_2O)]Cl_2\cdot 5MeOH\cdot H_2O$, and all of them in the crystals of Lu^{III} and Y^{III} compounds were not found in difference Fourier maps.

Figures presenting X-ray structures were made using the Mercury and Diamond programs.[10,11]

The details of crystal structures refinements are given in Table S1 and in the crystallographic information files (CIFs) deposited at the Cambridge Crystallographic Data Centre (CCDC Nos. 2112764–2112769).

Supporting Table S1. Experimental details for the crystals.

	$[La(L2^{R})Cl_3]\cdot 2.5MeOH\cdot 0.5H_2O$	$\{[Tb(L2^{R})Cl_2(MeOH)][Tb(L2^{R})Cl_2(H_2O)]\}Cl_2\cdot 9MeOH\cdot H_2O$	$[Lu_2(L1^{R})_2(\mu_2\text{-}F)_2(NO_3)_2](NO_3)_2\cdot CHCl_3\cdot 3H_2O$	$[Yb_2(L1^{R})_2(\mu_2\text{-}F)_2F(H_2O)]Cl_3\cdot 3.5MeOH\cdot 4.5H_2O$	$[Y_2(L3)_2(\mu_2\text{-}F)_2(NO_3)_2](NO_3)_2\cdot CHCl_3\cdot MeOH\cdot H_2O$	$[La_2(L2^{R})_2(\mu_2\text{-}F)_3F(H_2O)]Cl_2\cdot 5MeOH\cdot H_2O$
CCDC No.	2112764	2112765	2112766	2112767	2112768	2112769
Chemical formula	$C_{44.5}H_{45}Cl_3LaN_6O_3$	$C_{94}H_{112}Cl_6N_{12}O_{12}Tb_2$	$C_{53}H_{67}Cl_3F_2Lu_2N_{16}O_{15}$	$C_{55.5}H_{85}Cl_3F_3N_{12}O_9Yb_2$	$C_{38}H_{43}Cl_3F_2N_{16}O_{14}Y_2$	$C_{89}H_{92}Cl_2F_4La_2N_{12}O_7$
M_r	957.12	2132.49	1662.51	1573.78	1270.05	1866.46
Crystal system, space group	Triclinic, *P*1	Monoclinic, $P2_1$	Monoclinic, $P2_1$	Tetragonal, $P4_32_12$	Monoclinic, $P2_1/n$	Orthorhombic, $P2_12_12_1$
Temperature (K)	100	100	100	120	120	100
a, *b*, *c* (Å)	8.945(3), 9.554(3), 13.935(4)	18.086(2), 10.6576(13), 25.390(3)	14.746(2), 15.1265(14), 15.929(2)	19.041(2), 19.041(2), 36.640(5)	15.631(3), 14.302(3), 22.809(6)	16.572(2), 21.766(4), 25.180(5)
α, β, γ (°)	80.84(2), 77.81(2), 67.62(3)	90, 101.49(2), 90	90, 116.36(2), 90	90, 90, 90	90, 99.20(3), 90	90, 90, 90
V ($Å^3$)	1072.3(6)	4795.9(10)	3183.6(8)	13284 (3)	5033(2)	9083(3)
Z	1	2	2	8	4	4
Radiation type	Mo $K\alpha$	Mo $K\alpha$	Mo $K\alpha$	Mo $K\alpha$	Mo $K\alpha$	Mo $K\alpha$
μ (mm^{-1})	1.23	1.70	3.29	2.99	2.54	1.05
Crystal size (mm)	0.3 × 0.2 × 0.1	0.43 × 0.25 × 0.11	0.20 × 0.12 × 0.08	0.40 × 0.24 × 0.19	0.20 × 0.17 × 0.02	0.36 × 0.24 × 0.03
Diffractometer	Kuma KM4-CCD	Kuma KM4-CCD	Oxford Diffraction Xcalibur	Agilent Technologies Xcalibur R	Kuma KM4-CCD	Kuma KM4-CCD,
Absorption correction	Multi-scan	Analytical	Analytical	Analytical	Analytical	Multi-scan
T_{min}, T_{max}	0.683, 1.000	0.490, 0.828	0.587, 0.785	0.461, 0.560	0.585, 0.935	0.761, 1.000
No. of measured, independent and observed [$I > 2\sigma(I)$] reflections	9820, 6434, 6429	34288, 17279, 16656	20211, 10615, 10148	29316, 12781, 11274	28811, 9359, 4689	65341, 16881, 12961
R_{int}	0.050	0.024	0.025	0.038	0.161	0.108
$(\sin \theta/\lambda)_{max}$ ($Å^{-1}$)	0.703	0.676	0.606	0.617	0.606	0.606
$R[F^2 > 2\sigma(F^2)]$, $wR(F^2)$, S	0.042, 0.113, 1.05	0.026, 0.058, 1.04	0.027, 0.064, 1.05	0.041, 0.090, 1.09	0.075, 0.195, 0.94	0.084, 0.205, 1.12
No. of reflections	6434	17279	10615	12781	9359	16881
No. of parameters	554	1161	859	767	689	1105
No. of restraints	20	7	34	18	1	991
H-atom treatment	H-atom parameters constrained	H-atom parameters constrained	H-atom parameters constrained	H-atom parameters constrained	H-atom parameters constrained	H-atom parameters constrained
$\Delta\rho_{max}$, $\Delta\rho_{min}$ (e $Å^{-3}$)	1.03, −0.92	1.00, −0.57	1.52, −0.57	1.73, −0.91	0.75, −0.90	1.59, −1.77
Absolute structure parameter	0.007(14)	−0.038(4)	−0.011(4)	−0.026(5)	–	0.014(12)

Computer programs: CrysAlis PRO (Rigaku OD, 2020), CrysAlis PRO (Rigaku OD, 2018), SHELXT-2014 (Sheldrick, 2015), SHELXL2014/7 (Sheldrick, 2015).

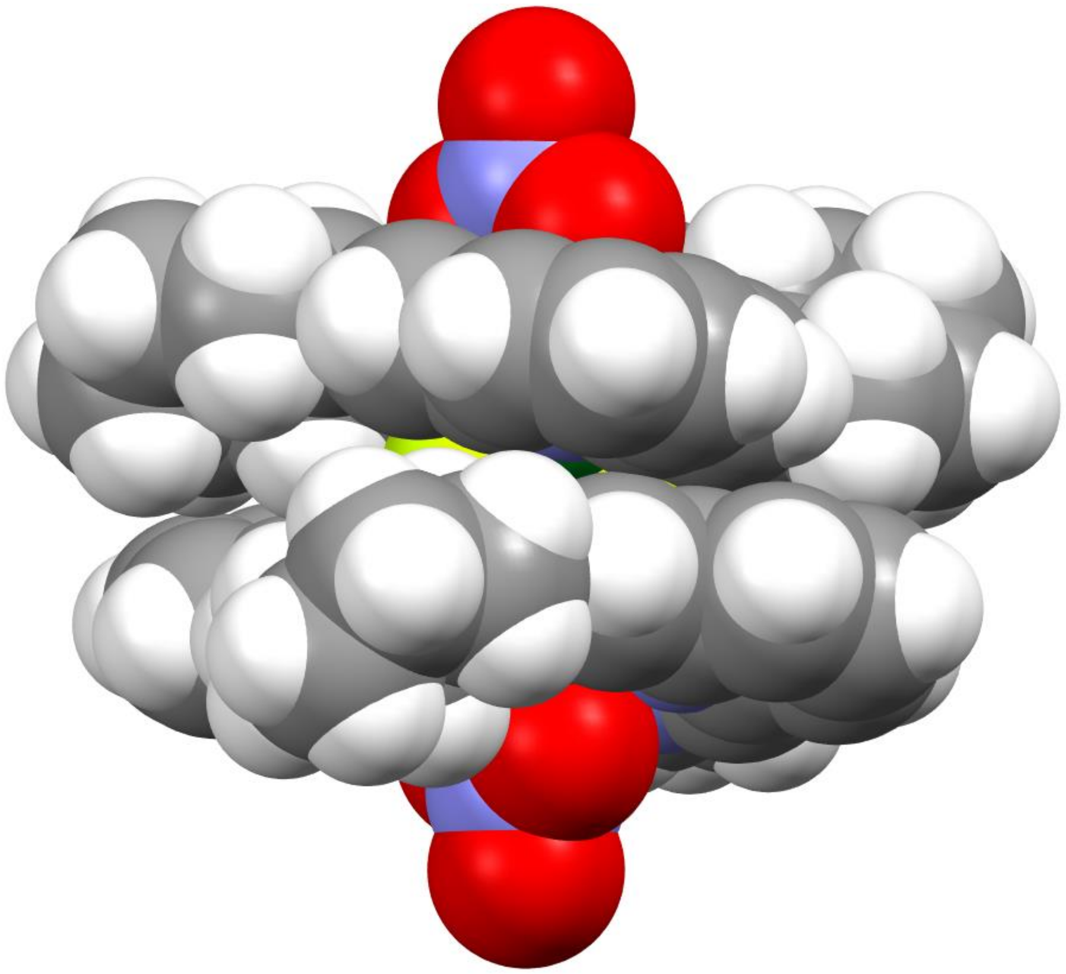

Supporting Figure S1. Side view of the dimeric cationic complex $[Lu_2(L1^R)_2(\mu_2\text{-}F)_2(NO_3)_2]^{2+}$ present in the crystal of $[Lu_2(L1^R)_2(\mu_2\text{-}F)_2(NO_3)_2](NO_3)_2 \cdot CHCl_3 \cdot 3H_2O$ in space fill representation.

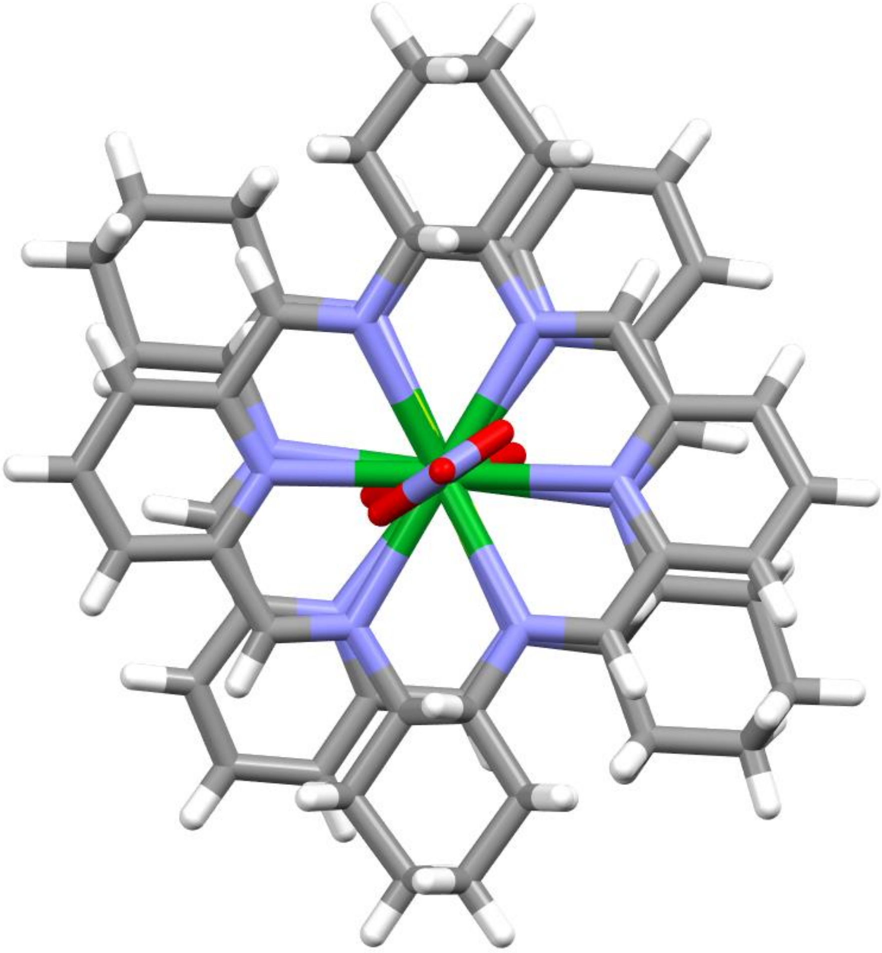

Supporting Figure S2. Top view of the dimeric cationic complex $[Lu_2(L1^R)_2(\mu_2\text{-}F)_2(NO_3)_2]^{2+}$ present in the crystal of $[Lu_2(L1^R)_2(\mu_2\text{-}F)_2(NO_3)_2](NO_3)_2\cdot CHCl_3\cdot 3H_2O$

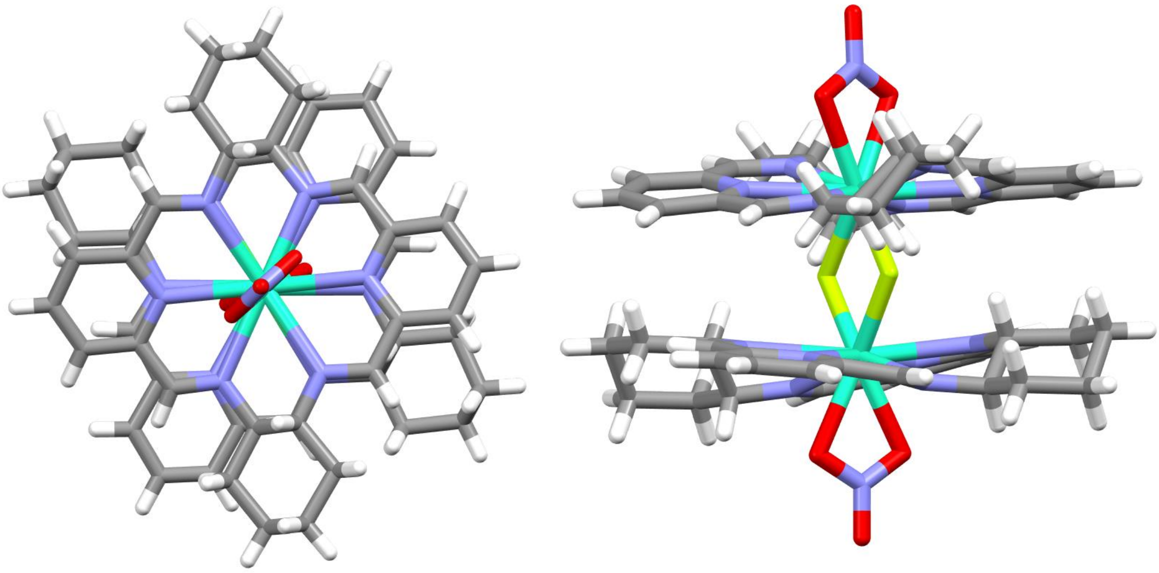

Supporting Figure S3. Top and side views of the dimeric cationic complex $[Dy_2(L1^{R})_2(\mu_2\text{-}F)_2(NO_3)_2]^{2+}$ in $[Dy_2(L1)_2(\mu_2\text{-}F)_2(NO_3)_2](NO_3)_2 \cdot CHCl_3 \cdot nH_2O$ crystal.

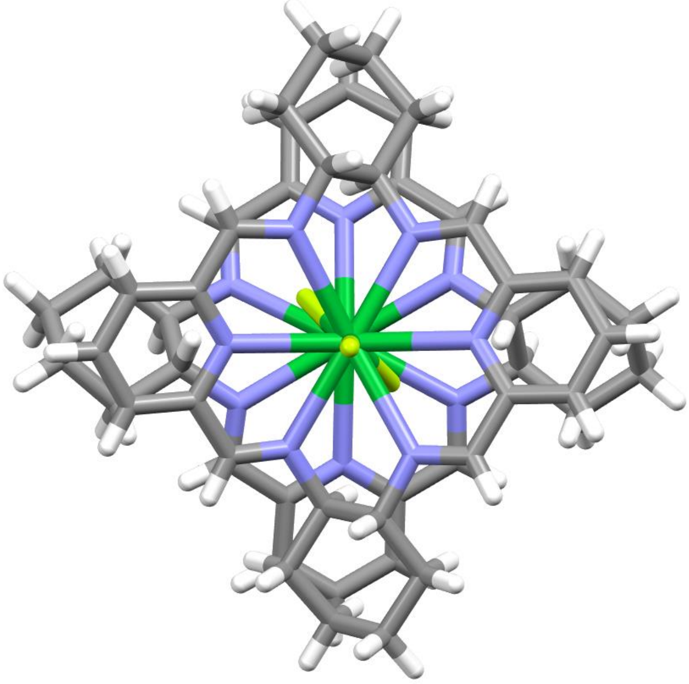

Supporting Figure S4. Top view of the dinuclear cationic complex $[Yb_2(L1^{R})_2(\mu_2\text{-}F)_2F(H_2O)]^{3+}$ present in the crystals of $[Yb_2(L1^{R})_2(\mu_2\text{-}F)_2F(H_2O)]Cl_3 \cdot 3.5MeOH \cdot 4.5H_2O$.

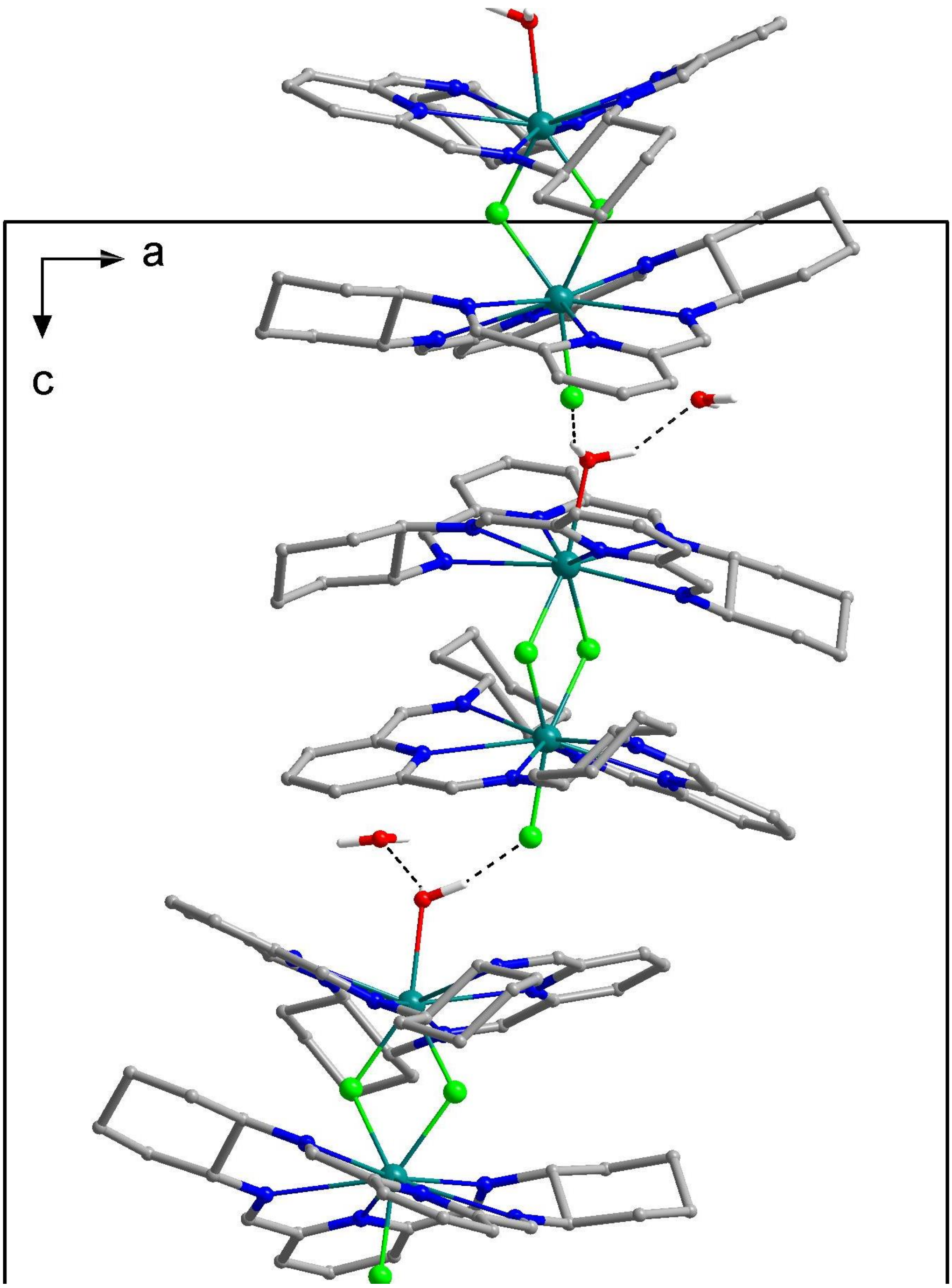

Supporting Figure S5. Helical chain formed by the $[Yb_2(L1^R)_2(\mu_2\text{-}F)_2F(H_2O)]^{3+}$ cations linked by O–H···F hydrogen bonds (hydrogen atoms other than those of water molecules omitted for simplicity).

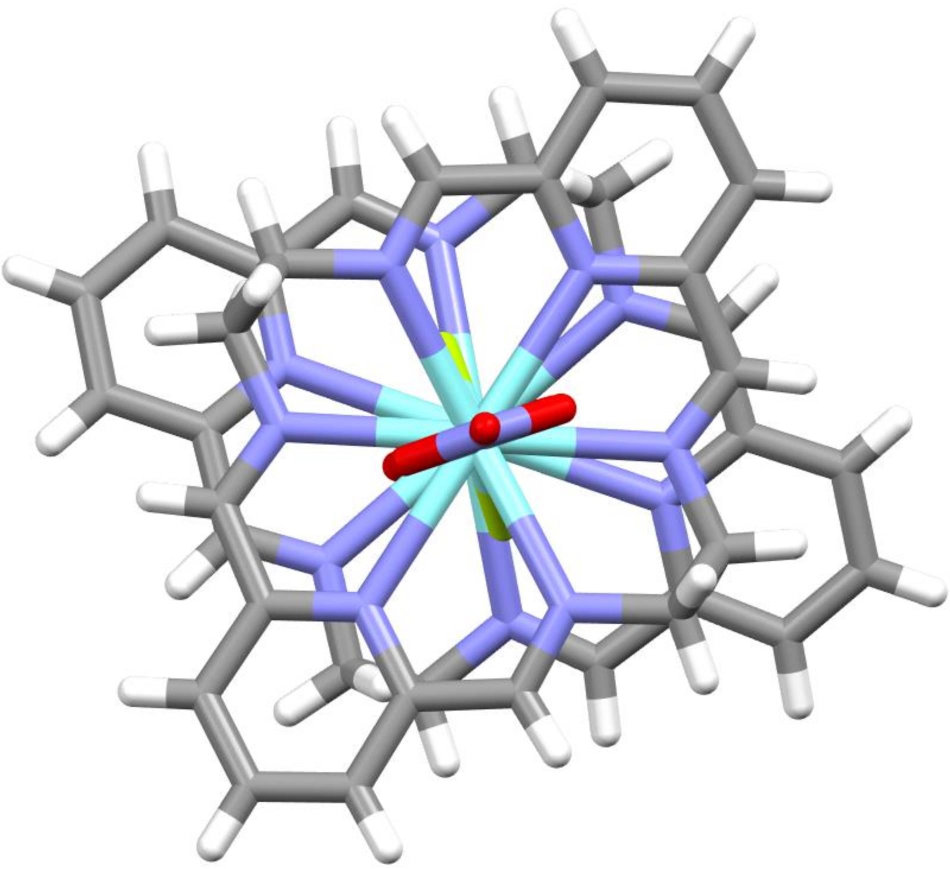

Supporting Figure S6. Top view of the dimeric cationic complex $[Y_2(L3)_2(\mu_2\text{-}F)_2(NO_3)_2]^{2+}$, present in the crystal of $[Y_2(L3)_2(\mu_2\text{-}F)_2(NO_3)_2](NO_3)_2\cdot CHCl_3\cdot MeOH\cdot H_2O$

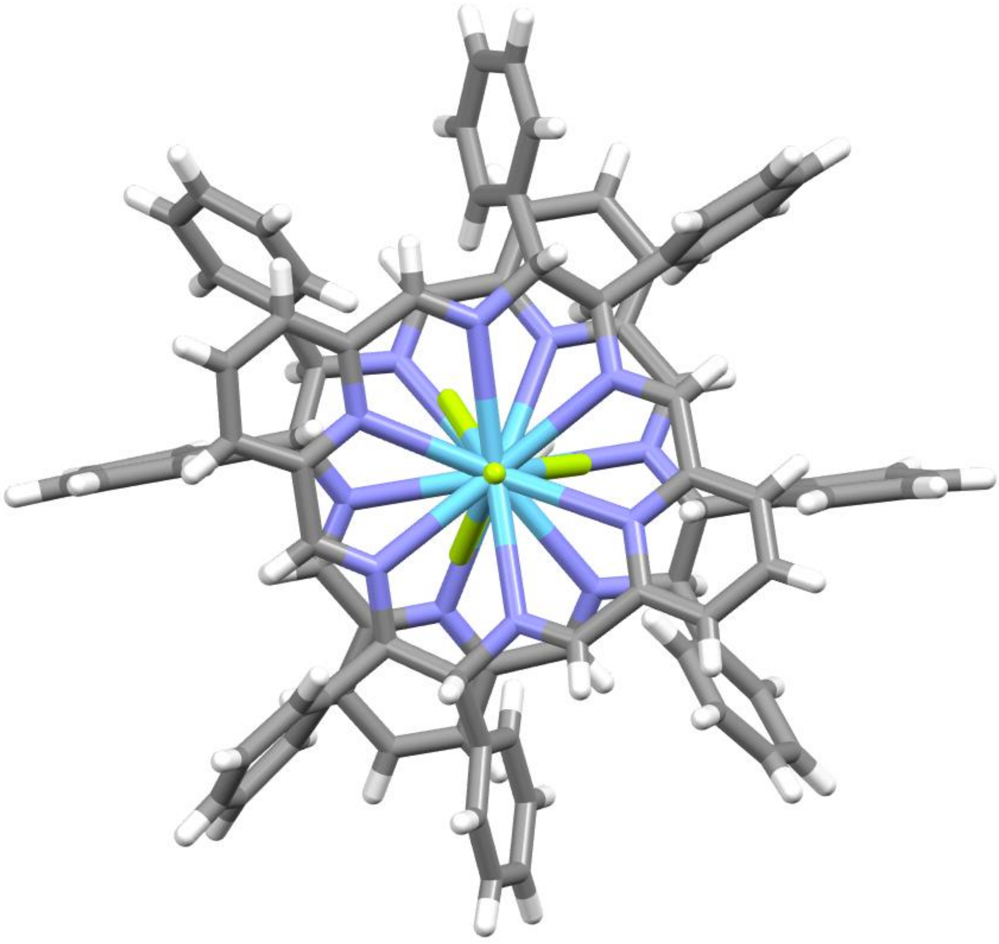

Supporting Figure S7. Top view of the $[La_2(L2^{R})_2(\mu_2\text{-}F)_3F(H_2O)]^{2+}$ cation present in the $[La_2(L2^{R})_2(\mu_2\text{-}F)_3F(H_2O)]Cl_2\cdot 5MeOH\cdot H_2O$ crystal.

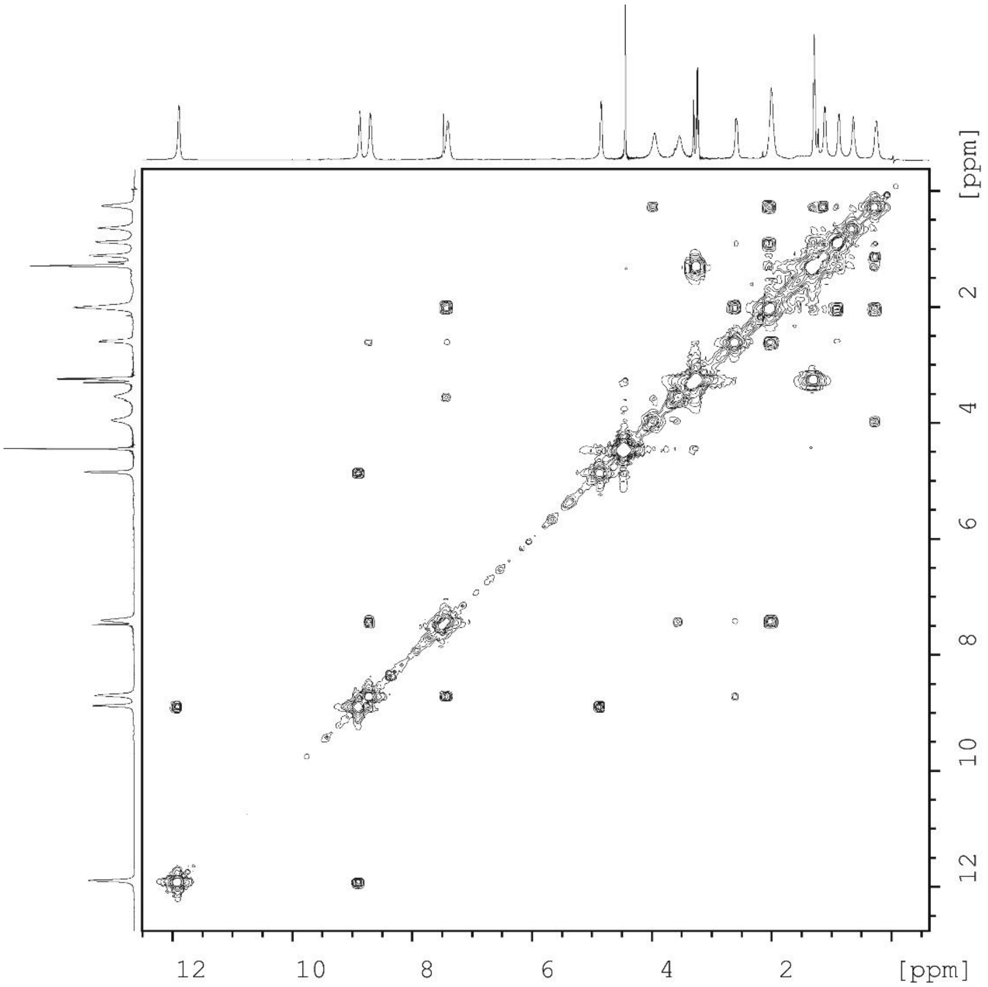

Supporting Figure S8. Region of the COSY spectrum ($CDCl_3/CD_3OD$ 2:1 v/v, 300 K) of the $[Eu_2(L1^{R})_2(\mu_2\text{-}F)_2(NO_3)_2](NO_3)_2\cdot 2H_2O$ complex.

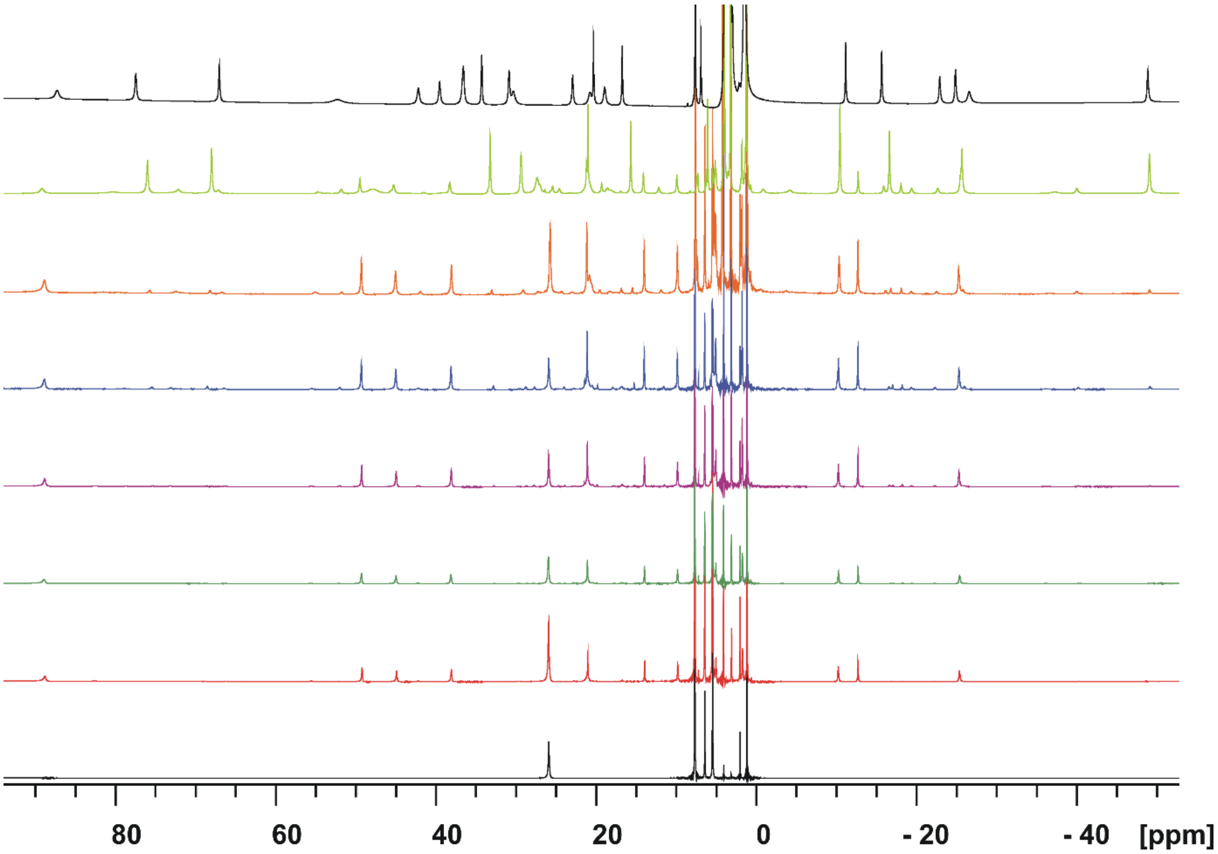

Supporting Figure S9. Region of the ^{1}H NMR spectra ($CDCl_3/CD_3OD$ 2:1 v/v, 300 K) of the $[Yb(L1^{R})(NO_3)_2](NO_3)$ complex and of the μ-fluoride dimers generated after addition of increasing amounts of NEt_4F. From bottom to top: 0, 0.5, 0.75 1, 1.5, 1.5 after 24h, 3 and 5 equivalents of NEt_4F, respectively

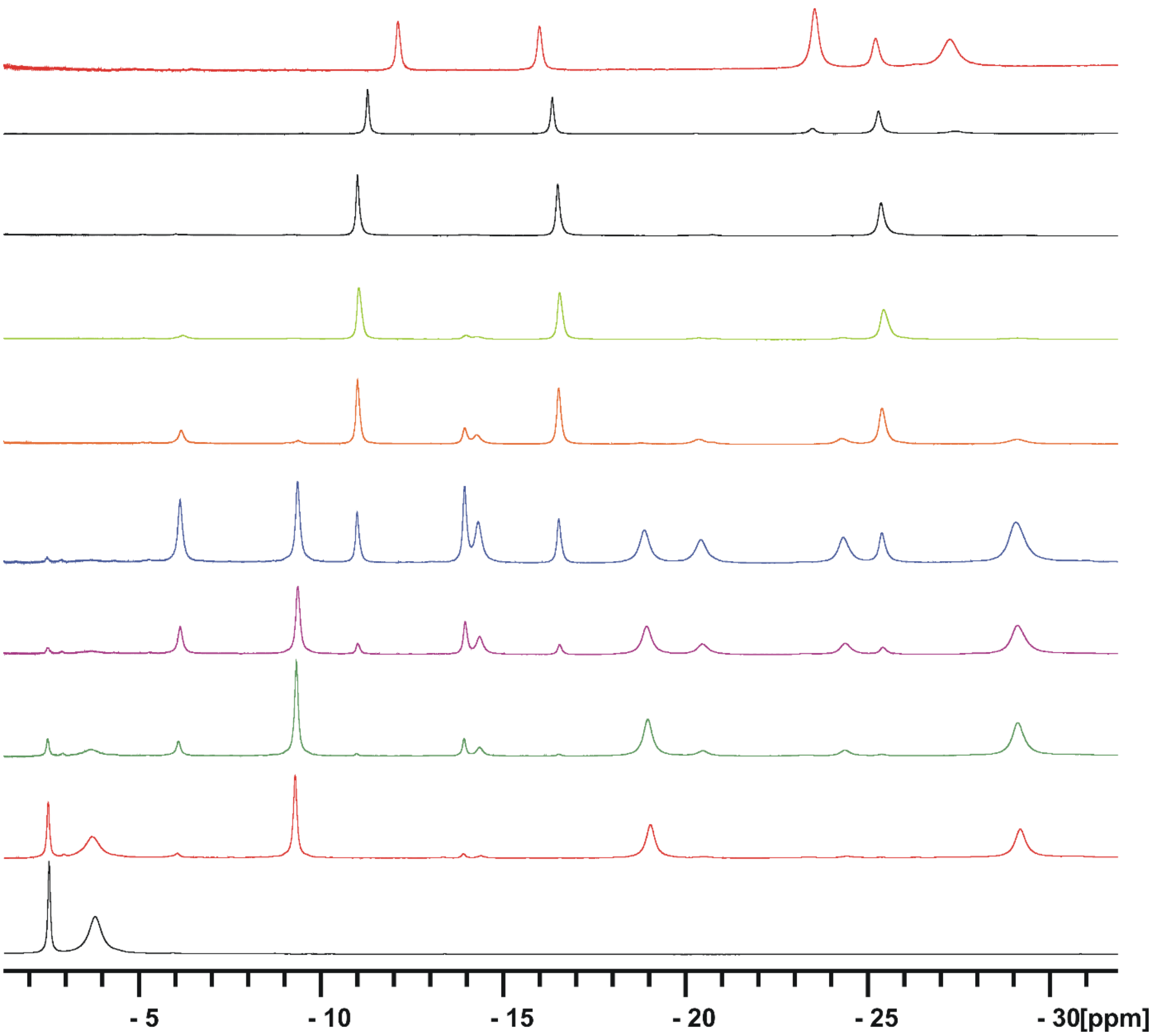

Supporting Figure S10. Region of the ^{1}H NMR spectra ($CDCl_3/CD_3OD$ 2:1 v/v, 300 K) of the [Yb(L1^{R})Cl_3] complex and of the μ-fluoride dimers generated after addition of increasing amounts of NEt_4F. From bottom to top: 0, 0.5, 0.8, 1, 1.3, 1.6, 1.8, 2, 4, and 10 equivalents of NEt_4F, respectively

References:

(1) Chang, V. Y.; Calvinho, K. U. D.; Tovar, R. C.; Johnson, V. A.; Straus, D. A.; Muller, G. Photophysical and Chiroptical Properties of the Enantiomers of N,N'-Bis(1-phenylpropyl)-2,6-pyridinecarboxamide and their Chiral 9-Coordinate Ln^{3+} Complexes. *Eur. J. Inorg. Chem.* **2020**, *40*, 3815-3828.

(2) (a) Muller, G. Luminescent chiral lanthanide(iii) complexes as potential molecular probes. *Dalton Trans.* **2009**, 9692-9707, (b) Muller, G. in *Luminescence of Lanthanide Ions in*

Coordination Compounds and Nanomaterials, Vol. First Edition (Ed.: A. de Bettencourt-Dias), John Wiley & Sons, Inc., Chichester, United Kingdom, **2014**, pp. 77-124. (c) MacKenzie, L. E.; Pålsson, L. O.; Parker, D.; Beeby, A.; Pal, R. Rapid time-resolved Circular Polarization Luminescence (CPL) emission spectroscopy. *Nat. Commun.* **2020**, *11*, 1676-1684.
(3) *CrysAlisPRO Software system*; Rigaku Oxford Diffraction: Oxford, UK, 2018, 2020.
(4) Mazurek, J.; Lisowski, J. Chiral macrocyclic lanthanide complexes derived from (1*R*,2*R*)-1,2-diphenylethylenediamine and 2,6-diformylpyridine. *Polyhedron* **2003**, *22*, 2877–2883.
(5) Li, Z. H.; Zhai, Y. Q.; Chen, W. P.; Ding, Y. S.; Zheng, Y. Z., Air-Stable Hexagonal Bipyramidal Dysprosium(III) Single-Ion Magnets with Nearly Perfect D_{6h} Local Symmetry. *Chem. Eur. J.* **2019**, *25*, 16219–16224.
(6) Sheldrick, G. M. Crystal structure refinement with *SHELXL*. *Acta Crystallogr., Sect. C: Struct. Chem.* **2015**, *71*, 3–8.
(7) Sheldrick, G. M. A short history of *SHELX*. *Acta Crystallogr., Sect. A: Found. Adv.* **2008**, *64*, 112–122.
(8) Sheldrick, G. M. *SHELXT* - Integrated space-group and crystal-structure determination. *Acta Crystallogr., Sect. A: Found. Adv.* **2015**, *71*, 3–8.
(9) Starynowicz, P.; Lisowski, J. Monomeric, dimeric and polymeric lanthanide(III) complexes of a hexaazamacrocyclic imine derived from 2,6-diformylpyridine and ethylenediamine. *Polyhedron*, **2015**, *85*, 232–238.
(10) *MERCURY*, ver. 3.8, Program for Crystal Structure Visualisation and Exploration, CCDC Cambridge University, 2016.
(11) Brandenburg, K. *DIAMOND*, version 3.2k. Crystal Impact GbR: Bonn, Germany, 2014.
